# Effectiveness of Low Emission Zones: Large Scale Analysis of Changes in Environmental NO_2_, NO and NO_x_ Concentrations in 17 German Cities

**DOI:** 10.1371/journal.pone.0102999

**Published:** 2014-08-12

**Authors:** Peter Morfeld, David A. Groneberg, Michael F. Spallek

**Affiliations:** 1 Institute for Occupational Epidemiology and Risk Assessment (IERA) of Evonik Industries, Essen, Germany; 2 Institute and Policlinic for Occupational Medicine, Environmental Medicine and Preventive Research, University of Cologne, Cologne, Germany; 3 Institute of Occupational Medicine, Social Medicine and Environmental Medicine, Goethe-University, Frankfurt am Main, Germany; 4 European Research Group on Environment and Health in the Transport Sector (EUGT), Berlin, Germany; The Ohio State University, United States of America

## Abstract

**Background:**

Low Emission Zones (LEZs) are areas where the most polluting vehicles are restricted from entering. The effectiveness of LEZs to lower ambient exposures is under debate. This study focused on LEZs that restricted cars of Euro 1 standard without appropriate retrofitting systems from entering and estimated LEZ effects on NO_2_, NO, and NO_x_ ( = NO_2_+NO).

**Methods:**

Continuous half-hour and diffuse sampler 4-week average NO_2_, NO, and NO_x_ concentrations measured inside and outside LEZs in 17 German cities of 6 federal states (2005–2009) were analysed as matched quadruplets (two pairs of simultaneously measured index values inside LEZ and reference values outside LEZ, one pair measured before and one after introducing LEZs with time differences that equal multiples of 364 days) by multiple linear and log-linear fixed-effects regression modelling (covariables: e.g., wind velocity, amount of precipitation, height of inversion base, school holidays, truck-free periods). Additionally, the continuous half-hour data was collapsed into 4-week averages and pooled with the diffuse sampler data to perform joint analysis.

**Results:**

More than 3,000,000 quadruplets of continuous measurements (half-hour averages) were identified at 38 index and 45 reference stations. Pooling with diffuse sampler data from 15 index and 10 reference stations lead to more than 4,000 quadruplets for joint analyses of 4-week averages. Mean LEZ effects on NO_2_, NO, and NO_x_ concentrations (reductions) were estimated to be at most −2 µg/m^3^ (or −4%). The 4-week averages of NO_2_ concentrations at index stations after LEZ introduction were 55 µg/m^3^ (median and mean values) or 82 µg/m^3^ (95th percentile).

**Conclusions:**

This is the first study investigating comprehensively the effectiveness of LEZs to reduce NO_2_, NO, and NO_x_ concentrations controlling for most relevant potential confounders. Our analyses indicate that there is a statistically significant, but rather small reduction of NO_2_, NO, and NO_x_ concentrations associated with LEZs.

## Introduction

Low Emission Zones (LEZs) are areas or roads where the most polluting vehicles are restricted from entering. They are currently introduced in 13 European countries [1]. In Europe, vehicle emissions are classified by the so-called “Euro Standards” with a current range from Euro 1 to Euro 6 regarding the technical features of the vehicles which are fixed in several EU-Directives for passenger cars and heavy-duty trucks [e.g., 2]. Basically, this means that vehicles are restricted in relation to their Euro emission level. The configuration of LEZs is extremely different and heterogeneous in Europe, for example in Italy, where the entry standards, the subsistent regulations and the daily duration of LEZ conditions differ substantially from town to town. However, most LEZs in Europe operate 24 hours a day, 365 days a year [see 1].

One of the most developed applications is found in Germany. Low emission zones have been introduced in Germany since 2008 in different stages, resulting in meanwhile 48 LEZs with restrictions for pollutant groups 2 or 3 in 11 Federal states by the end of January 2014 [3]. In this study we analysed the effect of introducing the “LEZ of pollutant group 1” which restricts from entering Diesel cars of an European emission standard below Euro 2 without particulate reduction system and gasoline cars of an European emission standard below Euro 1 without appropriate exhaust gas catalytic converters [3].

Traffic emissions are considered to be a relevant source of air pollution [4] and LEZs are believed to be the most effective measure that cities can take to reduce vehicle-induced air pollution problems in their area [5]–[7]. The emissions that are aimed to be reduced by LEZs are mainly fine particles like PM10 or smaller [8]–[12]. The effectiveness of LEZs to reduce traffic-related exposures is still under debate [13] and there is an open discussion in the public about the “outcome” and cost-benefit ratio of LEZs [14]–[16]. Most of the published information refers to particulate matter.

Additionally, nitrogen dioxide is discussed to be a major traffic-related pollutant as well as an epidemiologic marker of air quality and related adverse health effects [17]–[21]. On the other hand, a systematic literature review showed only moderate evidence for adverse health effects at a long-term exposure below an annual mean of 40 µg/m^3^ NO2 [22].

According to EU rules [23], [24] limits were additionally imposed for NO2 and are enforced in Germany since 2010: 200 µg/m^3^ as an 1 hour average (acceptable: 18 excursions/year) and 40 µg/m^3^ as an annual average. Values were and are in excess: about 69% of all stations near to traffic showed annual averages higher than 40 µg/m^3^ in Germany [7], [25]. This non-compliance is not restricted to Germany but the European limit value for NO2 is exceeded in many European cities [26]–[28]. The LEZ concept was extended and it was assumed that LEZs are an effective measure not only to lower PM10 dust levels but also to reduce NO2 concentrations [6], [29]. There are indications that LEZs may indeed reduce NOx concentrations effectively [30]–[32], but ozone has to be considered a confounder in NO2 measurements [e. g. 33], and the gases NO and NO2 rapidly interconvert, too [34]. Furthermore, national emission ceilings were defined for NOx, i.e., the sum of NO2 and NO [35]. Thus, there is interest in the impact of LEZs on concentrations of NO and NOx also [36].

However, a scientific proof of the LEZ concept targeting at NO_2_, NO, and NO_x_ is still missing. In order to test the views of legislators and researchers that LEZs are effective measures to reduce nitrogen oxide concentrations [29], [37], this study focused on the potential effects of LEZs on ambient concentrations of NO_2_, NO, and NO_x_ in LEZ areas of 17 German cities.

We reported on the effect of LEZs on PM_10_ concentrations elsewhere [32].

## Methods

### Target parameters

The aim of the study was to analyse the effectiveness of German LEZs (as many as eligible) to lower NO_2_, NO, and NO_x_ ( = NO_2_+NO) concentrations. The first analysis series of NO_2_, NO, and NO_x_ were based on continuous half-hour measurement data of NO_2_ and NO. Second, measurement data for NO_2_ and NO concentrations collected by diffuse samplers and determined over longer sampling periods were available. These data were allocated to 4-week periods. Third, we collapsed the half-hour measurement data to four-week averages and pooled these collapsed continuous data and the diffuse sampler data to perform joint analyses over 4-week periods. The original NO_2_ and NO measurements were performed by the Environmental State Institutions in Germany (Landesumweltämter). A federal data base [38] reports on the applied measurement procedures.

### Measuring procedure

Two measuring procedures were applied: continuous measurement devices (chemiluminescence), data stored as half-hour averages and diffuse samplers (Palmes tubes, chromatography), data stored as long-term averages over weeks. The chemiluminescence method relies on the reaction of NO with O3: NO+O3→NO2*+O2. Chemiluminescence is generated in the range of 600 nm to 3,000 nm when the excited molecules return to the ground state. The light intensity is proportional to the concentration of NO molecules. A deoxidation converter is used to reduce NO2 to NO. Thus, the NO2 concentration is determined as the difference between the NOx concentration measured when the sample gas is directed through a deoxidation converter and the NO concentration measured when the gas is not run through the converter. The diffuse samplers were Palmes type tubes modified with a glass frit as turbulence barrier. In these passive samplers molecules diffuse because of a concentration gradient through an intake opening with a defined cross-section along a fixed diffusion path to a sampling medium by which they are adsorbed. This process is described by Fick's first diffusion law. The chemical analysis is done by chromatography. [More details on both methods may be found in 39], [40]–[43].

### Period of investigation

The period of investigation was from 2005 until the end of 2009 (31 December 2009), starting at least from the introduction of the individual LEZ minus the length of the respective LEZ phase (or earlier if restrictions of truck traffic were enforced before the introduction of the LEZ).

### Low Emission Zones

There were 34 German active LEZs until the end of 2009 and 774 monitoring stations in use. With introduction of these LEZs, as a main effect, only those diesel vehicles with an exhaust emission standard better than Euro 1 (with sticker) were allowed to enter the zone. In principle, the German “LEZ of pollutant group 1” restricts from entering

Diesel passenger cars, trucks and buses of an European emission standard below Euro 2 without particulate reduction system, and
Gasoline passenger cars, trucks and buses of an European emission standard below Euro 1 without appropriate exhaust gas catalytic converters.

Local authorities can set up exception permits especially for light duty vehicles, trucks and buses due to local necessities [3].

According to protocol LEZs were included into the study if and only if

monitoring stations existed, that operated before and after the LEZ introduction and measured inside the LEZ area (*index stations*) and
monitoring stations existed, that operated before and after the LEZ introduction and measured outside the LEZ area – in a circle around the centre with a radius of about 25 km – and if outside the city area, than in no other LEZ (*reference stations*) and
these monitoring stations measured NO_2_ or NO (continuous measurements or diffuse samplers).

(For the terminology and the use of index and reference values in comparisons if exposures levels see Rothman et al. [44])

**Figure 1:**
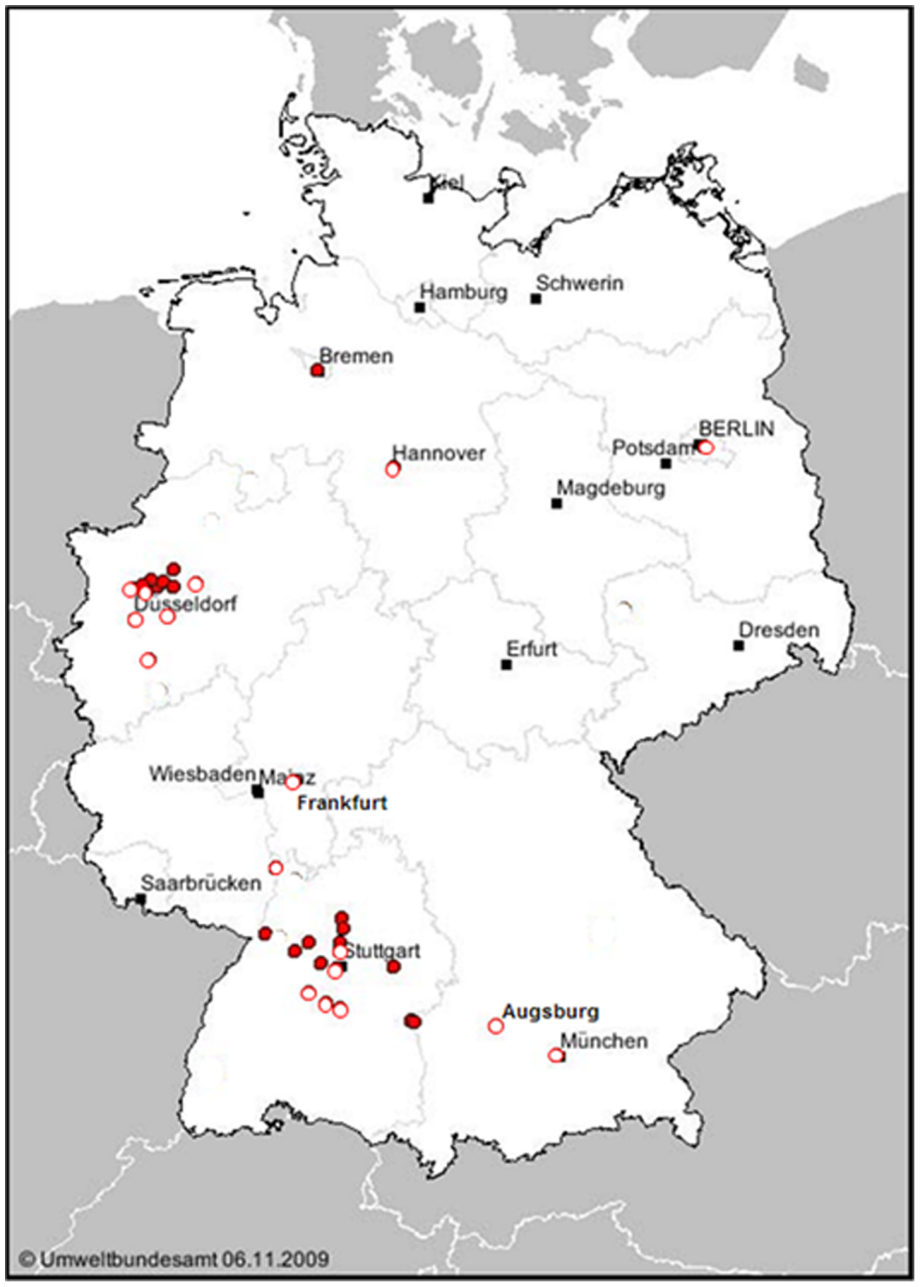
Active and investigated LEZs in Germany, December 2009. The 17 LEZs included into the study are marked with open red circles. The 17 LEZs that were active but were excluded according to protocol are indicated by full red circles. Capital cities of the Federal states are shown by black squares. This is a modification of the map as published at URL www.umweltbundesamt.de/umweltzonen. Date of access: 6th November 2009.

Seventeen cities with LEZs in 6 German Federal states could be included into the study (Baden-Württemberg: Herrenberg, Ludwigsburg, Mannheim, Reutlingen, Stuttgart, Tübingen; Bavaria: Augsburg, Munich; Berlin: Berlin; Hesse: Frankfurt; Lower Saxony: Hannover; North Rhine-Westphalia: Dortmund, Duisburg, Düsseldorf, Essen, Cologne, Wuppertal). Figure 1 shows all active 34 German active LEZs in December 2009 and the 17 LEZs included for study. File S1 entails maps of all LEZs eligible for study with all index and reference stations marked (Figure S1 in S1 to Figure S19 in S1).

In total, these 17 LEZs, eligible for study, contained 108 eligible monitoring stations with 53 index stations and 55 reference stations. The data base constructed from transferred data encompassed a total of 9,517,911 data lines which were used as input to analysis. An overview is given in Table 1.

**Table 1:** Overview of low emission zones (LEZs) in German cities included for analysis: 17 LEZs in 6 federal states.

LEZ				Measurement Stations Suitable For Analysis									
		Data Since	Introduction of LEZ	NO_2_		NO		NO_x_		Area LEZ/km^2^	Area City/km^2^	Population LEZ	Population City
Federal State	City			Index	Reference	Index	Reference	Index	Reference			2007	2011
Baden-Württemberg^a^	Herrenberg	2006	01Jan2009	1	1^d^	1	1^d^	1	1^d^	17	64.71	15000	29935
	Ludwigsburg	2005	01Mar2008	2	1^d^	2	1^d^	2	1^d^	30	43.33	55000	86939
	Mannheim	2005	01Mar2008	2	2	2	2	2	2	7.5	144.96	93900	291458
	Reutlingen	2004	01Mar2008	1	2^d^	1	2^d^	1	2^d^	4.5	87.06	24500	110084
	Stuttgart	2004	01Mar2008	5	2^d^	5	2^d^	5	2^d^	207	207	590000	591015
	Tübingen	2005	01Mar2008	1	1^d^	1	1^d^	1	1^d^	11.9	108.12	64000	83248
Total Baden-W^a^				12	9	12	9	12	9	277.9	655.18	842400	1192679
Bavaria^a^	Augsburg	2007	01Jul2009	3	1	3	1	3	1	5.2	146.93	40000	272699
	Munich	2006	01Oct2008	5	3	5	3	5	3	44	310.71	431000	1388308
Total Bavaria^a^				8	4	8	4	8	4	49.2	457.64	471000	1661007
Berlin “BLUME”^a^	Berlin	2004	01Jan2008	5	9	5	9	5	9	88	891.85	1100000	3375222
Hesse^a^	Frankfurt a.M.	2003	01Oct2008	1	3	1	3	1	3	110	248.31	550000	687775
Lower Saxony^a^	Hannover	2005	01Jan2008	1	4	1	4	1	4	50	204.14	218000	514137
North Rhine-Westphalia^a^	Dortmund	2006	01Jan2008	2	4^d^	2	4^d^	2	4^d^	19.1	280.71	ca. 158000	572087
	Duisburg	2006	01Oct2008	2	1	2	1	2	1	ca. 50	232.83	ca. 376500	486816
	Düsseldorf	2006	15Feb2009	1	3	1	3	1	3	13.8	217.41	36500	593682
	Essen	2006	01Oct2008	3	2^d^	3	2^d^	3	2^d^	84	210.34	ca. 291000	566862
	Cologne	2006	01Jan2008	2	4	2	4	2	4	16	405.17	130000	1023373
	Wuppertal	2006	01Feb2009	1	2^d^	1	2^d^	1	2^d^	35.3	168.39	194000	342885
Total North Rhine-W^a^				11	16	11	16	11	16	218.2	1514.85	1186000	3585705
Total (contin) a				38	45	38	45	38	45	793.3	3971.97	4367400	11016525
Berlin “RUBIS”^b^	Berlin	2004	01Jan2008	8	9	8	9	8	9	88	891.85	1100000	3375222
North Rhine-Westphalia^b^	Essen	2006	01Oct 2008	7	1	-	-	-	-	84	210.34	ca.291000	566862
Total (diffuse)^b^				15	10	8	9	8	9	172	1102.19	1391000	3942084
Total (overall)^c^				53	55	46	54	46	54	965.3	5074.16	5758400	14958609

Number of index and reference stations per gaseous component (NO_2_, NO, NO_x_) suitable for analysis of the first tier of LEZs until Dec 31, 2009.

a continuous measurements, half-hour concentration averages.

b diffuse samplers, longer sampling periods (over weeks).

c all data combined (continuous, diffuse), adjusted to concentration averages over 4-weeks periods.

d reference station used more than once in comparisons to index stations.

### Data analysis

The data set structured for analysis consisted of matched quadruplets. A matched quadruplet comprises four pairwise corresponding measurement values consisting of two index- and two reference values. One index value and the simultaneously measured reference value were obtained during the active LEZ period, the other pair of values was obtained before introducing the LEZ. The pairs of values had a 364 days difference in time of or a multiple of 364 days, hence keeping the season, day of the week and time of day constant within the quadruplets. The allocation of reference stations to index stations was done pairwise, i.e., quadruplets were constructed by the data of one index station and allocating to it all appropriate reference stations with their data without a prior collapsing (“collapsing” is a technical term widely used in statistics describing the summary of a table in marginal, http://www.stata.com/manuals13/dcollapse.pdf). The method has been described in detail before [45] and is a refined approach in comparison to other analytical strategies [46]. The analysis plan was critically reviewed by a chair of statistics.

The quadruplets were analysed by the “difference score method in the two period case” [47]: Differences in index values were regressed on differences in reference values while other data were taken into account as covariates in fixed-effect regression analyses. Two types of models were fitted: a linear (additive) model and a log-linear (multiplicative) model. The difference of the index concentration data was used as the response variable in the linear model. The log of this response variable was entered into the log-linear regression model after applying an appropriate positive offset calculated from the data [48]. The two model types differ in the assumption on how covariables may influence the index station concentration data: on an additive scale or on a multiplicative scale [49], [50].

The following covariables were taken into account in the basic fixed effects regression analyses: differences at reference stations in µg/m^3^ (to control e.g. for large-scale meteorological changes and seasonal effects), baseline data at reference stations in µg/m^3^ (to control for time-dependent effects of reference data, Allison [47], and baseline data at index stations in µg/m^3^ (to control for “regression to the mean” [51]. This structure defines the basic regression approach. The covariables were entered into the log-linear (multiplicative) models after adding an appropriate offset if indicated [48] and then taking logs.

**Figure 2:**
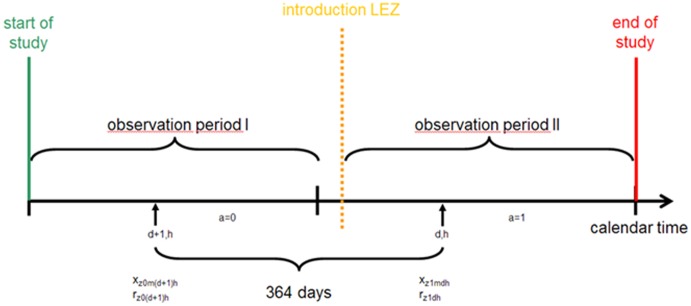
Index (x_zamdh_) and reference concentration (r_zadh_) at index measurement station m and LZ z in observation period II with active LEZ (a = 1) and in observation period I with inactive LEZ (a = 0): matched quadruplets consisting of two index measurement values and two reference measurement values. The time difference between compared measurement values at day d of the year in period 2 and d+1 of the year in period 1, always with starting time h, is not a full year but 364 days to keep the weekday constant.

The following equation describes the analysis of matched quadruples in the basic fixed-effect linear (“additive”) regression model [47]

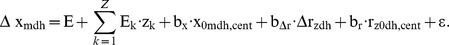
Δ x_mdh_ describes the difference of the index station data at monitoring station m between days d and d-364 ( = day d+1 in the year before), always at time (hour) h, i.e., x_1mdh_ - x_0mdh_ (compare Figure 2). x_0mdh,cent_ denotes the baseline value at station m on day d at time h, centred at the mean of all baseline values at station m. The terms Δr_dh_ and r_z0dh,cent_ are the corresponding reference value data. The coefficient of major interest is the intercept of the regression model because it estimates the LEZ effect: E measures the mean effect across all LEZs, E+E_k_ the mean effect in zone k, 1≤k≤Z. The coefficient b_x_ accounts for “regression to the mean”, b_Δr_ for the bias in annual levels (e.g., changed meteorological conditions), b_r_ for a time-dependent effect of reference values and ε is the residual error of the concentration difference at the index stations. The second model type had the same structure but used logs of the terms (“log-linear”, “multiplicative”). An appropriate small offset was added to avoid undefined logarithms [48].

The equation of the basic fixed-effect linear (“additive”) regression model can be justified as follows (to keep the notation simple we suppress the time index: we write, eg, x_z0_ instead of x_z0dh_).

Let us start with assuming an ideal hypothetical situation: measurements are without any distortions and random errors, no covariates are operating. In that case we will measure the index concentration at time point 0 before the LEZ was introduced as a constant value c_0_ at all index stations of the LEZ. After the introduction of the LEZ we will measure at time point 1 at the same index station the constant value c_1_. The effectiveness of the zone is simply E = c_1_−c_0_.

But even if there are no biases, random errors and no covariates we do not expect to see the same E for all LEZs. The effectiveness may depend on characteristics of the zone k like the area of the LEZ, A_k_ (e.g., we may expect a larger effect if the LEZ area is larger). The concentration at time point 1 may be written more appropriately as c_1_+f×A_k_ (with a multiplicative coefficient f mapping the effect of the area into the concentration scale). The effect of zone k can be described as E = c_1_+f×A_k_−c_0_. Note that A_k_ operates as an effect modifier.

We can take account of differences between the zones without referring to a specific characteristic of LEZ k, like the area: We may describe the effect of zone k in more abstract terms as E+E_k_ [E+E_k_ means E+E_k_×z_k_ with a multiplicative indicator z_k_, that takes the value 1 for zone k and zero otherwise, 1≤k≤Z]. E_k_ ist the specific effect offset of LEZ k in comparison to the overall mean E of the LEZ effects. It is simple to extend the notation to cover different baseline concentrations for the different LEZs. Thus, E+E_k_ = c_1_+(c_z1_−c_1_)−[c_0_+(c_z0_−c_0_)] = c_z1_−c_z0_, i.e., the effect of zone k is the difference between the zone specific measurement values after (c_z1_) and before (c_z0_) the introduction of the LEZ k (c_1_ and c_0_ now denote the averages of the concentrations across all LEZs under study).

Still, the approach is not very realistic. We should take into account background variations of the intensities, resulting from e.g. large area changes of the concentrations. These large area variations are reflected in the values r_z0_ and r_z1_ at the reference stations. Despite all efforts to measure the concentrations as precisely as possible we always will have random errors ε_1_ and ε_0_. In this extended approach the measurement values for zone k before introducing the LEZ are x_z0_ = c_0_+(c_z0_−c_0_)+g×r_z0_+ε_0_ and x_z1_ = c_1_+(c_z1_−c_1_)+g×r_z1_+ε_1_ after the introduction. The factor g measures how strong the reference values do influence the index values. It follows that x_z1_−x_z0_ = E+E_k_+g* (r_z1_−r_z0_)+(ε_1_−ε_0_). With Δ x_z_ = x_z1_−x_z0_, Δ r_z_ = r_z1_−r_z0_, ε = ε_1_−ε_0_ and b_Δr_ = g we yield the major part of the equation of the basic fixed-effect linear (“additive”) regression model. Note that we substituted z by m which means that we apply the approach in a refined way to every index monitoring station m. We have demonstrated above that a potential confounder/adjuster, like the concentration at a reference station, enters the equation in terms of the difference of the values across time (e.g., Δ r_z_ = r_z1_−r_z0_). And we have seen that the model can be extended by potential modifiers of the LEZ effect (“interaction terms”), like the area, by adding terms like f×A_k_ (in contrast to adjusters not as a difference in time). We will now explain in more detail why we included the effect modifying variables x_z0_ and r_z0_ additionally.

Altman and Bland [52] and Bland and Altman [53] suggested including the mean value x_zm_ of the concentrations at index station m as another covariate: this allows the difference Δ x_zm_ to depend on the average concentration at the station. This inclusion of x_zm_ operates again a distortion due to “regression to the mean” [54]. This phenomenon is inevitably complicating longitudinal comparisons. Baseline values that are very high due to random errors will probably not be reproduced but lower values will be measured, and this is so even if the null hypotheses of no effect is true [51], [55]–[57]. A better strategy to correct for this potential distortion is to include x_z0m,cent_, i.e., the baseline values at the index station [58], [59]. Including additionally r_z0, cent_ was exercised in Allison [47], p. 10. This approach allows for a flexible adjustment of the annual level bias because we get rid of the assumption of a time-invariant effect of the reference station values on the index stations values. The covariates x_z0m, cent_ and r_z0, cent_ are centered on the mean of the values of each measuring station so that the terms E und E_k_ can be interpreted without further transformations.

Since the impact of meteorological conditions is extremely relevant [e.g., 60], the following data were collected to be used in addition to the reference station data to control for distortions due to meteorological changes. We took over the height of the inversion base H in m, the wind velocity V in m/s, the amount of precipitation P in mm/h from the PAREST project for all investigated measurement stations and half hours in the follow-up period [61]–[65].

We extended the basic regression models to the regression model 1 approach by adjusting additionally for the change of the three meteorological variables at the index stations According to the box model of meteorology [66] the differences were calculated on the additive scale after transforming the variables into 1/H, 1/(V+0.1 m/s), and 1/(P+0.1 mm/h). The smallest unit of scale was 0.1 throughout, hence this value was used as an offset to avoid divisions by zero [48]. Differences were determined on the multiplicative scale after taking logs of these terms [48]. We adjusted for the time span (in years) between measurements considered within a quadruplet in order to adjust for trends in concentration levels before the LEZ was introduced. In multiplicative models log of the time span was used after applying an appropriate offset [48].

In regression model 2 approach, the following time-dependent binary indicators were additionally adjusted for: period of school holidays (yes/no), period of environmental bonus paid (yes/no) and periods when trucks were not allowed to enter the area where the measurement station was located (yes/no). In Germany a bonus was paid to car owners between January 14, 2009 and November 2, 2009 if they bought a new car with a reduced exhaust emission (http://www.bafa.de/bafa/de/wirtschaftsfoerderung/umweltpraemie/index.html). These binary indicators were entered also into the extended log-linear (multiplicative) models.

This statistical approach was successfully validated in advance to the study in an analysis of simulated data from FU Berlin [67]. The simulated data was produced by the PAREST project [61], [63, www.parest.de]. The major aim of this project was the identification of emission reducing strategies by simulation. Transport and distribution models were developed and applied, the so called REM-CALGRID approach [68]–[70]. The model was applied to the city of Munich, and simulated half-hour PM_10_ data were generated for each of the five index and three reference stations (see Figure S10 in File S1). Data of the year 2005 were simulated twice, with and without adding an LEZ effect (the value of the imprinted effect was unknown to the analyzing working group). 280,320 data lines were transferred. The simulated PM_10_ concentrations were analyzed and showed a mean value of about 21 µg/m^3^ at the index stations and 18 µg/m^3^ at the reference stations. The basic additive regression model estimated an LEZ effect of −0.130 µg/m^3^, the multiplicative model a relative change of −0.7%. The PAREST research report [63, www.parest.de] described the LEZ effect that was imprinted: PM_10_ mean values are reduced in Munich city by at most 0.2 µg/m^3^ or at most 1%.

Additive and multiplicative regression models were fitted to sub-sets of the data to perform sensitivity analyses: continuous measurement data, continuous measurement data collapsed to four-week averages, diffuse sampler data, pooled diffuse sampler and collapsed continuous data, always with and without excluding times with restrictions of truck traffic; quadruplets produced by index traffic stations only. The pooled continuous and diffuse sampler measurement data determined for four weeks periods was of major interest in this study because the annual average is the most critical endpoint to consider (see Introduction section) and these data cover both types of measurement data. Because annual data are generally too coarse for LEZ effect estimation, we followed-up on averages over about a month. The additive and multiplicative regression models analysing these sets of data were specified with three sets of covariables as described above. All basic models, the models evaluating continuous data collapsed to 4-week averages and the models fitted to single index stations were not used for statistical testing of the LEZ effects. Tests for effects measured by NO_2_, NO, and NO_x_ quadruplets were not considered as independent. According to this structure, we evaluated 2*3*2*2*2 = 48 statistical tests for each of the three endpoints. Due to this multiple testing scenario we applied an adapted significance level of 5%/50 = 0.1% [“family wise error rate”, 71].

We fitted additionally explorative models that estimated the size of the LEZ effect at each index station enrolled. In addition, we estimated mean effects of the LEZs across the Federal states. The results of these exploratory analyses were mainly used for internal discussions of the project steering committee (see Acknowledgement).

All regression models used robust estimators of coefficient variances. All data analyses were performed using Stata 11 [72] on a 64-bit PC.

## Results

### NO_2_ - continuous measurements

The basic data consisted of 6,412,864 data lines leading to 3,038,781 quadruplets of continuous NO_2_ measurement (half-hour averages) from 6 Federal states and 17 LEZs with 38 index stations and 45 reference stations. Table 2 gives an overview of the distributions observed: on average, NO_2_ concentrations were between 50 µg/m^3^ and 52 µg/m^3^ at the index stations and between 26 µg/m^3^ and 27 µg/m^3^ at the reference stations. The differences at the stations varied substantially in a range of hundreds of µg/m^3^ upwards and downwards. A comparison of mean and median differences at index and reference stations indicated a crude LEZ effect estimate of about −1 µg/m^3^. In the linear model 1 the absolute effect estimate was similar: −1.11 µg/m^3^ (Table 3). The model 1 results showed a time-dependent impact of reference station data, a pronounced “regression to the mean”, a clear influence of the three meteorological variables (independently from the crude adjustment by reference station data), and a downward trend of concentrations before the LEZs were introduced. The direction of impact of the meteorological variables was as expected: the smaller H, V, or P the larger the index NO2 concentrations. In linear model 2 the LEZ effect estimate was slightly more pronounced: −1.85 µg/m3. In the log-linear model 1 (multiplicative approach), the relative effect estimate was 0.979, i.e., a reduction of 2.1% was found (Table 4). The estimated impact of covariables agreed with the finding in the corresponding linear model. When applying regression model 2 the relative LEZ effect estimate was 0.961, i.e., the reduction was estimated to be 3.9%.

**Table 2:** NO_2_: Quadruplets of continuous NO_2_-measurements: index stations (Ind), reference stations (Ref) before (pre) and after (post) introduction of LEZ.

Statistic	Ind,pre	Ind,post	Ref,pre	Ref,post	Ind.diff.	Ref.diff
N	3038781	3038781	3038781	3038781	3038781	3038781
min	0.4	1.3	0.4	0.5	−330	−215
p5	12	11	4.0	4.0	−52	−33
p50	45.0	43.8	20.7	20.6	−1.0	0.0
mean	51.959	50.831	26.383	26.17	−1.128	−0.212
p95	115	114	68	67	49	33
max	392	436	248	434	375	317

Ind.diff and Ref.diff denote differences between index measurements and between reference measurements (negative post-pre differences indicate lower values after introduction of LEZ). Concentrations measured in µg/m^3^.

N: number of quadruplets; Min: minimum, p5: 5^th^ percentile, p50: median, mean: arithmetic average, p95: 95^th^ percentile, max: maximum, unit:

**Table 3:** NO_2_: Linear (additive) model 1 evaluating the quadruplets of continuous NO_2_-measurements.

Ind.diff	Coef.	Std. Err.	t	p	95% Conf.	Interval
Ref.diff	0.677	0.001	623	<0.001	0.675	0.679
Ref.base	0.509	0.001	417	<0.001	0.507	0.512
Ind.base	−0.644	0.001	−845	<0.001	−0.645	−0.642
Diff 1/H	564	2.8	200	<0.001	558	569
Diff 1/V	3.12	0.025	126	<0.001	3.07	3.17
Diff 1/P	0.088	0.004	23.6	<0.001	0.081	0.095
Time.diff	−0.399	0.023	−17.7	<0.001	−0.443	−0.354
E	−1.112	0.013	−87.3	<0.001	−1.137	−1.087

Regression coefficient, robust standard errors of coefficient, t-statistic, two-sided P-value, and 95%-confidence interval of coefficient. The absolute LEZ effect estimate is given by the coefficient E in µg/m^3^ (<0: concentration is lowered by LEZ).

Covariables: difference in reference stations Ref.diff in µg/m^3^, centered reference baseline concentration Ref.base in µg/m^3^, centered index baseline concentration Ind.base in µg/m^3^, Diff 1/H = difference in 1/(height of inversion layer) in 1/m, Diff 1/V = 1/(wind velocity+0.01) in (m/s)^−1^, difference in 1/P = 1/(amount of precipitation+0.01) (mm/h)^−1^, centered difference in time Time.diff in years.

**Table 4:** NO_2_: Log-linear (multiplicative) model 1 evaluating the quadruplets of continuous NO_2_-measurements.

ln Ind.diff	Coef.	Std. Err.	t	p	95% Conf.	Interval
ln Ref.diff	0.296	0.001	582	<0.001	0.295	0.297
ln Ref.base	0.265	0.002	141	<0.001	0.262	0.269
ln Ind.base	−0.981	0.002	−412	<0.001	−0.986	−0.977
Diff ln 1/H	0.071	0.001	194	<0.001	0.070	0.071
Diff ln 1/V	0.12	0.001	301	<0.001	0.119	0.121
Diff ln 1/P	0.014	0.001	29	<0.001	0.013	0.015
ln Time.diff	−0.025	0.001	−32	<0.001	−0.027	−0.024
ln E	−0.021	0.0	−77	<0.001	−0.022	−0.021

Regression coefficient, robust standard errors of coefficient, t-statistic, two-sided P-value, and 95%-confidence interval of coefficient. The relative LEZ effect estimate is given by the coefficient E (<1: concentration is lowered by LEZ).

relative effect E = 0.979, 95% Conf. Interval = 0.979, 0.980.

Covariables, before taking logs: difference in reference stations Ref.diff in µg/m^3^, centered reference baseline concentration Ref.base in µg/m^3^, centered index baseline concentration Ind.base in µg/m^3^, 1/H = 1/(height of inversion layer) in 1/m, 1/V = 1/(wind velocity+0.01) in (m/s)^−1^, 1/P = 1/(amount of precipitation+0.01) (mm/h)^−1^, centered difference in time Time.diff in years.

### NO_2_ - pooled continuous and diffuse sampler measurement data

6,133 data lines and 4,095 quadruplets of NO_2_ pooled continuous and diffuse sampler measurement data (averaging period: four weeks) were examined from 17 LEZs with 53 index stations and 55 reference stations. A crude comparison based on the observed distributions revealed a LEZ effect of about −0.2 µg/m^3^ to −0.6 µg/m^3^ (Table 5). Using the linear model 1 approach the absolute effect estimate was −0.826 µg/m^3^ (Table 6). The meteorological variables showed no substantial impact due to the long averaging period. Model 2 estimated the LEZ effect as −1.73 µg/m^3^. The log-linear modelling led to a relative effect of 0.980 (Table 7, model 1) or 0.961 (model 2). Table S1 in File S1 provides a detailed overview of the results when fitting a series of models to analyse the NO_2_ measurements. LEZ effect estimates were about −1 µg/m^3^ to −2 µg/m^3^ (additive models) or −2% to −4% (multiplicative models).

**Table 5:** NO_2_: Quadruplets of pooled continuous and diffuse sampler NO_2_-measurements: index stations (Ind), reference stations (Ref) before (pre) and after (post) introduction of LEZ.

Statistic	Ind,pre	Ind,post	Ref,pre	Ref,post	Ind.diff.	Ref.diff
N	4095	4095	4095	4095	4095	4095
min	13	11	6	5	−32	−23
p5	26	24	11	10	−14	−13
p50	57.31	54.23	41.04	39.78	−2.34	−1.71
mean	56.298	54.246	40.007	38.18	−2.053	−1.824
p95	84	82	66	64	12	8
max	134	136	75	76	41	25

Ind.diff and Ref.diff denote differences between index measurements and between reference measurements (negative post-pre differences indicate lower values after introduction of LEZ). Concentrations measured in µg/m^3^.

N: number of quadruplets, Min: minimum, p5: 5^th^ percentile, p50: median, mean: arithmetic average, p95: 95^th^ percentile, max: maximum.

**Table 6:** NO_2_: Linear (additive) model 1 evaluating the quadruplets of pooled continuous and diffuse sampler NO_2_-measurements.

Ind.diff	Coef.	Std. Err.	t	p	95% Conf.	Interval
Ref.diff	0.564	0.018	31.7	<0.001	0.529	0.599
Ref.base	0.394	0.025	15.9	<0.001	0.345	0.442
Ind.base	−0.695	0.019	−36.7	<0.001	−0.732	−0.657
Diff 1/H	−65.1	20.8	−3.13	0.002	−106	−24.3
Diff 1/V	0.034	0.148	0.23	0.816	−0.256	0.325
Diff 1/P	0.009	0.028	0.30	0.764	−0.047	0.064
Time.diff	0.369	0.189	1.95	0.051	−0.001	0.739
E	−0.826	0.123	−6.71	<0.001	−1.068	−0.585

Regression coefficient, robust standard errors of coefficient, t-statistic, two-sided P-value, and 95%-confidence interval of coefficient. The absolute LEZ effect estimate is given by the coefficient E in µg/m^3^ (<0: concentration is lowered by LEZ).

Covariables: difference in reference stations Ref.diff in µg/m^3^, centered reference baseline concentration Ref.base in µg/m^3^, centered index baseline concentration Ind.base in µg/m^3^, Diff 1/H = difference in 1/(height of inversion layer) in 1/m, Diff 1/V = 1/(wind velocity+0.01) in (m/s)^−1^, difference in 1/P = 1/(amount of precipitation+0.01) (mm/h)^−1^, centered difference in time Time.diff in years.

**Table 7:** NO_2_: Log-linear (multiplicative) model 1 evaluating the quadruplets of pooled continuous and diffuse sampler NO_2_-measurements.

ln Ind.diff	Coef.	Std. Err.	t	p	95% Conf.	Interval
ln Ref.diff	0.359	0.015	23.9	<0.001	0.329	0.388
ln Ref.base	0.024	0.005	4.49	<0.001	0.014	0.034
ln Ind.base	−0.039	0.004	−10.1	<0.001	−0.047	−0.032
Diff ln 1/H	−0.016	0.003	−5.32	<0.001	−0.022	−0.01
Diff ln 1/V	0.013	0.003	4.21	<0.001	0.007	0.02
Diff ln 1/P	0.011	0.003	3.68	<0.001	0.005	0.017
ln Time.diff	0.005	0.006	0.95	0.34	−0.006	0.017
ln E	−0.02	0.003	−7.82	<0.001	−0.025	−0.015

Regression coefficient, robust standard errors of coefficient, t-statistic, two-sided P-value, and 95%-confidence interval of coefficient. The relative LEZ effect estimate is given by the coefficient E (<1: concentration is lowered by LEZ).

relative effect E = 0.980, 95% Conf. Interval = 0.975, 0.985.

Covariables, before taking logs: difference in reference stations Ref.diff in µg/m^3^, centered reference baseline concentration Ref.base in µg/m^3^, centered index baseline concentration Ind.base in µg/m^3^, 1/H = 1/(height of inversion layer) in 1/m, 1/V = 1/(wind velocity+0.01) in (m/s)^−1^, 1/P = 1/(amount of precipitation+0.01) (mm/h)^−1^, centered difference in time Time.diff in years.

### NO - pooled continuous and diffuse sampler measurement data

A total of 5,790 data lines from 17 LEZs with 46 index stations and 54 reference stations were available to analyse pooled continuous and diffuse sampler NO measurement data. A descriptive analysis of the 4,005 quadruplets indicated a LEZ effect of about 0 µg/m^3^ to −1 µg/m^3^ (Table 8). Using the additive approach the absolute effect estimate was −1.13 µg/m^3^ in model 1 (Table 9). When the model specification 2 was applied the LEZ effect estimate changed the sign: +0.38 µg/m^3^, i.e., no reduction was indicated in this extended model type. The log-linear regression model of type 1 yielded a relative effect estimate of 0.968 (Table S2 in File S1). The direction of the estimated relative effect changed when model 2 was applied: +1.20.

**Table 8:** NO: Quadruplets of pooled continuous and diffuse sampler NO-measurements: index stations (Ind), reference stations (Ref) before (pre) and after (post) introduction of LEZ.

Statistic	Ind,pre	Ind,post	Ref,pre	Ref,post	Ind.diff.	Ref.diff
N	4005	4005	4005	4005	4005	4005
min	2.7	2.5	1.0	1.0	−86	−50
p5	4.6	4.4	2.2	2.2	−22	−21
p50	48.54	43.44	31.83	26.32	−2.59	−1.26
mean	49.479	46.373	34.153	31.025	−3.105	−3.129
p95	95	95	86	81	17	13
max	230	251	139	104	77	56

Ind.diff and Ref.diff denote differences between index measurements and between reference measurements (negative post-pre differences indicate lower values after introduction of LEZ). Concentrations measured in µg/m^3^.

N: number of quadruplets, Min: minimum, p5: 5^th^ percentile, p50: median, mean: arithmetic average, p95: 95^th^ percentile, max: maximum.

**Table 9:** NO: Linear (additive) model 1 evaluating the quadruplets of pooled continuous and diffuse sampler NO-measurements.

Ind.diff	Coef.	Std. Err.	t	p	95% Conf.	Interval
Ref.diff	0.666	0.022	30.5	<0.001	0.623	0.708
Ref.base	0.496	0.026	19.1	<0.001	0.445	0.547
Ind.base	−0.473	0.022	−21.5	<0.001	−0.516	−0.430
Diff 1/H	75.8	29.5	2.57	0.010	18.0	134
Diff 1/V	−0.571	0.216	−2.65	0.008	−0.994	−0.148
Diff 1/P	0.028	0.048	0.59	0.557	−0.066	0.123
Time.diff	0.245	0.313	0.78	0.433	−0.369	0.860
E	−1.128	0.218	−5.19	<0.001	−1.555	−0.702

Regression coefficient, robust standard errors of coefficient, t-statistic, two-sided P-value, and 95%-confidence interval of coefficient. The absolute LEZ effect estimate is given by the coefficient E in µg/m^3^ (<0: concentration is lowered by LEZ).

Covariables: difference in reference stations Ref.diff in µg/m^3^, centered reference baseline concentration Ref.base in µg/m^3^, centered index baseline concentration Ind.base in µg/m^3^, Diff 1/H = difference in 1/(height of inversion layer) in 1/m, Diff 1/V = 1/(wind velocity+0.01) in (m/s)^−1^, difference in 1/P = 1/(amount of precipitation+0.01) (mm/h)^−1^, centered difference in time Time.diff in years.

### NO_x_ - pooled continuous and diffuse sampler measurement data

The analysis of pooled continuous and diffuse sampler NO_x_ measurement data was performed using 4,005 quadruplets that originated from a set of 5,790 data lines generated by 46 index stations and 54 reference stations of 17 LEZs. According to the distributions of differences a crudely estimated LEZ effect (based on averages or medians) was present of about −0.2 µg/m^3^ to −1.3 µg/m^3^ (Table S3 in File S1). Adjusting for covariables in linear model 1 returned an absolute effect estimate of −1.74 µg/m^3^ (Table S4 in File S1). The adjustment for further covariables (regression model 2) led to an effect estimate of −0.89 µg/m^3^. When the log-linear model 1 was used (Table S5 in File S1), a relative LEZ effect of 0.976 was found. The adjustment for additional covariates (model 2) led to a change in direction: the relative effect was estimated as 1.048.

### Summary of Results for NO_2_, NO, and NO_x_

Table 10 gives an overview of the findings for NO_2_, NO, and NO_x_. The mean concentration levels at the index stations were about 50 µg/m^3^ for NO_2_ and for NO, thus, about 100 µg/m^3^ for NO_x_. Model 1 analyses showed reductions of the concentrations after introducing the LEZs. Although small, all effect estimates were statistically significant at the 0.1% level. Model 1 estimates based on an additive structure gave compatible findings to the log-linear multiplicative approach (e.g., 2% of 50 µg/m^3^ = 1 µg/m^3^). The model 1 LEZ effect estimates were similar to, but slightly more pronounced than crude LEZ effect estimates based on direct comparisons of the measurement differences at index stations and reference stations within the quadruplets while ignoring the impact of covariables (compare Tables 2, 5, and 8 and Table S1 in S1). All analyses point to the conclusion that on average the concentration reducing effect of LEZs was smaller than 2 µg/m^3^ for each of the three components NO_2_, NO, and NO_x_, i.e., not higher than about 4%, when considering all investigated index stations. However, breaking down the analyses by Federal states or LEZs yielded heterogeneous estimates of effects.

**Table 10:** Summarized Results on Nitrogen Oxides.

			Model 1 Estimates		Model 2 Estimates	
	Evaluation Period	Mean Index Concentration/µg/m^3^	Additive Effect/µg/m^3^	Multiplicative Effect/%	Additive Effect/µg/m^3^	Multiplicative Estimate/%
NO_2_	0.5 h	50	−1.1	−2.1	−1.9	−3.9
	4 weeks	55	−0.8	−1.9	−1.7	−3.9
NO	4 weeks	48	−1.1	−3.2	+0.4	+1.9
NO_x_	4 weeks	103	−1.7	−2.4	−0.9	+4.8

Model 1 covariables: difference in reference stations Ref.diff in µg/m^3^, centered reference baseline concentration Ref.base in µg/m^3^, centered index baseline concentration Ind.base in µg/m^3^, Diff 1/H = difference in 1/(height of inversion layer) in 1/m, Diff 1/V = 1/(wind velocity+0.01) in (m/s)^−1^, difference in 1/P = 1/(amount of precipitation+0.01) (mm/h)^−1^, centered difference in time Time.diff in years.

The NO_2_ analysis was based on 192 comparisons of index vs reference stations, among them were 31 index stations characterized as “background”, one characterized as “industry” and 160 as “traffic” stations. We performed a sensitivity analysis by restricting the evaluation to the stations close to traffic. The additive linear type 2 model estimated an effect of −1.73 µg/m^3^ at all index stations (see last line in Table S1 in S1). When the analysis only accounted for the traffic stations we got a slightly more pronounced LEZ effect estimate of −2.26 µg/m^3^ (3,406 quadruplets, pooled data: four week averages). An analysis of the continuous data yielded almost the same result: −2.35 µg/m^3^ (2,105,702 quadruplets, half-hour averages).

## Discussion

In this study we analysed the effect of introducing the “Low Emission Zone (LEZ) of pollutant group 1” (which restricts from entering Diesel cars of an European emission standard below Euro 2 without particulate reduction) on NO_2_, NO, and NO_x_ concentrations in Germany. We included as many LEZs as possible (17 out of 34 in 2009 met our inclusion criteria) into a homogeneous analysis of nitrogen oxide data measured before and after the introduction of LEZs of pollutant group 1 until the end of 2009. We used matched quadruplets of index and reference station values and analysed the changes in concentrations with fixed-effect regression models while adjusting for important covariables. We performed sensitivity analyses by applying two model structures (additive and multiplicative) with varying sets of covariables to different subsets of the data. We based our study on precisely matched quadruplets to avoid distortions and to increase validity. A potential downside of the increased validity is a loss in precision due to the reduced data set eligible for analysis. However, the loss in power was negligible in this application because P-values were small even when taking multiple testing into account [73]. The statistical approach was successfully validated in advance to the study in an analysis of simulated data from FU Berlin [67]. We checked whether the adjustment in one model that analyzed all LEZs simultaneously and assumed unknown but identical covariate coefficients was appropriate for all LEZs. To do so we evaluated each LEZ separately and performed a meta-analysis on the findings. The precision weighted mean of the effect estimates at all index stations (n = 192, −1.71 µg/m^3^) was almost identical to the overall additive linear type 2 model (−1.73 µg/m^3^, see last line in Table S1 in S1). We conclude that the fitted single model that evaluated all LEZs simultaneously was appropriate and did not suffer from an insufficient adjustment.

As an overall finding the average effect of LEZ introduction on nitrogen oxide concentrations (NO_2_, NO, and NO_x_ = NO_2_+NO) was not higher than 2 µg/m^3^ at all index stations, i.e., not higher than about 4%. The effect was only slightly larger when we restricted the analyses to stations close to traffic. In the main analyses the coefficients describing the reductions were statistically significant on the 0.1% level, i.e., after taking multiple testing into account. We note, however, that the P-values calculated are potentially too small because autocorrelations in the data were not taken into account.

We detected a substantial heterogeneity of effects across the investigated LEZs and Federal states. However, this finding is not surprising because

the realisation of LEZs differed between states and within states (e.g., date of introduction, covered population and area of LEZs differ (compare Table 1), some operate together with an additional restriction of van traffic)
the degree of representativeness of monitoring stations inside the LEZs differs across LEZs (index stations: distances from centre/border of LEZ differ, used as background or hot spot stations and sometimes placed in street canyons)
the degree of representativeness of monitoring stations outside the LEZs differs across LEZs (reference stations: distances from LEZ differ, traffic conditions differ)
the applied measuring systems differ (continuous chemiluminescense procedure vs diffuse long-term sampling with chromatography).

The large variation of LEZ effect estimates across the LEZs should be put into perspective by considering the phenomenon of “regression-to-the-mean” [51]. Due to this phenomenon we expect that single observations with high baseline values show potentially decreasing trends – and low baseline values potentially increasing trends. This is true even under the null hypothesis of no causal LEZ effects on nitrogen oxide concentrations. “Regression-to-the-mean” has been shown to be rather pronounced in this study. Thus, the interpretation of single LEZs effect estimates is clearly limited and we will not report any details with the consent of the involved state institutions who performed the measurements (see Acknowledgements).

Models of type 2 showed more instability and returned positive effect estimates in some situations (see Table 10). Regression model 2 included as additional variables time-dependent binary indicators for period of school holidays, period of environmental bonus paid and periods when trucks were not allowed to enter the area where the measurement station was located. In some LEZs these variables were highly correlated with the active LEZ periods so that unstable findings due to collinearities can be expected. Such collinearities can introduce a bias away from the null and may generate exaggerated negative or positive model coefficients even if the true effects are near to zero [74]. Log-linear models showed to be more sensitive to these distortions. This may indicate a less appropriate modelling of the data when assuming multiplicative effects of covariates.

There are evaluations available concerning potential effects of LEZs on NO_2_ concentrations summarized by the German Federal Environmental Agency [7]: A total NO_2_ reduction by 5% and a local traffic-related NO_2_ reduction by 12% may be reached given “LEZ of pollutant group 3” so that only cars with a green sticker (Diesel vehicles of Euro 6, 5, 4 or Euro 3 with particle filter, gasoline cars with catalytic converter) are allowed to enter the LEZ [3]. This statement is based mainly on preliminary evaluations of the Berlin LEZ data by Rauterberg-Wulff and Lutz [29]. Puls and Jäger-Ambrozewicz [75] reported for the Frankfurt LEZ and an observation period until the end of 2011 effects of less than 3% which is closer to our present findings although they also cover a period of “LEZ of pollutant group 2” after Jan1, 2010. Only cars with a yellow sticker (Diesel vehicles of Euro 3 or 4 standard or Euro 2 with particle filter, gasoline cars with catalytic converter) were allowed to enter the Frankfurt LEZ after Jan 1, 2010 [3]. Bruckmann et al. [6] reported reductions of the annual average of NO_2_ concentrations up to 2% associated with the introduction of “LEZs of pollutant group 1” in North-Rhine Westphalia, and an absolute LEZ effect of about −1.2 µg/m^3^. In Hannover no NO_2_ reduction could be shown after introducing an “LEZ of pollutant group 1” [76]. All of these statements, however, were based on crude comparisons without sufficiently adjusting for important covariates like weather conditions, and traffic restrictions etc. Only Puls and Jäger-Ambrozewicz [75] applied a more sophisticated approach. They performed a time-series analysis and fitted regression models for the Frankfurt LEZ. These models', however, were not correctly specified as they did not include differences of the covariables but the absolute values only, and so they could not control for potential confounding effects although this was intended by the authors. All publications cited above reported only on individual LEZs or certain Federal states in Germany and not on the LEZ effect on the national level. Generalisations from these data are problematic because of the heterogeneous configurations of LEZs. A realistic estimate should be based on a homogeneous analytical approach covering as many LEZs and Federal states simultaneously as possible, as performed in this study.

Table 11 presents an overview of other study results published in the peer-reviewed literature on forecasted or measured LEZ effects on NO_2_ concentrations.

**Table 11:** Overview of studies estimating the effect of LEZs on NO_2_ concentrations.

Study	Country	City/Area	Intervention	NO_2_ effect/µg/m^3^	Comments
Builtjes et al. 2012,	Germany	Berlin	LEZ of level 3: forbidden<Euro 4	−1.3	
Stern 2013		Munich		−1.0	
		Ruhr Area		−1.7	
Tonne et al. 2008,	UK	London	Congestion Charging Zone: forbidden<Euro 4	−0.64 (−1.1%)	simulation study 2008
Kelly et al 2011				reduction up to −7.3%	simulation study 2011
Briggs 2008	Italy	Rome	2 LEZs:	−2.3/−3.0	simulation study: main effect due to the exclusion of Euro 0 vehicles
			Euro 0 forbidden 2 LEZs:		
Cesaroni et al. 2012	Italy	Rome	forbidden<Euro 4	−3.0/−4.1	
Boogaard et al. 2012	The Netherlands	Amsterdam	LEZ:	−4.5	analysis of measurements: crude comparisons, no covariates taken into account
		Den Bosch	trucks forbidden	(not statistically significant)	
		The Hague	<Euro 2 and Euro 3 trucks only allowed if retrofitted		
		Tilburg			
		Utrecht			
Johansson et al. 2009	Sweden	Stockholm	Road Pricing System: Vehicles travelling into and out of the charge cordon were charged for every passage during weekdays	NO_x_:	analysis of measurements: crude comparisons, no covariates taken into account
				−0.23 (Greater Stockholm)	
				−0.81 (inner city)	
This study	Germany	17 cities	LEZ of level 1: Diesel passenger cars, trucks and buses forbidden<Euro 2 without particulate reduction system	NO_2_, NO, NO_x_:	analysis of measurements: construction of matched quadruplets, regression analyses with covariates, all estimated effects statistically significant at the 0.1% level
				reduction less than −2.0 (−4%)	
				[PM_10_: reduction less than −0.2 µg/m^3^ (−1%)]	

Our results are in good accordance with the prognosis study PAREST of FU Berlin [61], [63]. An extensive description of the project is available [77]. The prognoses of PAREST are comparable with our estimates at all index stations because PAREST worked with an area coarseness defined by grid square of about 1 km×1 km and, thus, cannot estimate changes at single stations. Duyzer et al. [78] studied whether monitoring station data are representative for the population living in the area and concluded that the background station data are more appropriate to describe the impact on the citizens than the hot spot traffic stations. We conclude that the findings of PAREST and our results about the effect at all index stations should be preferred in an evaluation (not the effect estimates restricted to the traffic stations). PAREST predicted LEZ effects on NO_2_ levels assuming that only cars with green stickers are allowed to enter (LEZ of pollutant level 3). For the Berlin LEZ the authors calculated a reduction of about 1 µg/m^3^ to 1.3 µg/m^3^ in the city centre (relative: 3% to 5%), for the Munich LEZ a reduction of 1 µg/m^3^ in the city centre (relative: up to 5%), for the Ruhr area a reduction of 1 µg/m^3^ to 1.7 µg/m^3^ (relative: 3% to 4%). Setting the whole Ruhr area to a LEZ of pollutant level 3 lead to the prognosis of a reduction in NO_2_ concentrations of 1 µg/m^3^ to 2 µg/m^3^ (relative: 3% to 6%). It needs to be taken into account that these prognoses by PAREST are based on the pollutant level 3 LEZ scenario. We do not expect, therefore, that our findings from this study may change relevantly if the LEZs are extended to cover larger areas or if stricter traffic restrictions are applied.

A very large LEZ was introduced in London as a congestion charging zone. However, only prognoses of the potential LEZ effect on nitrogen oxide concentrations are available. NO_x_ reductions between 3.8% in 2008 up to 7.3% in 2012 along roadways were predicted in a modelling scenario for the London LEZ with vehicles and buses required to meet Euro 4 standards compared to current LEZ restrictions for Euro 3 vehicles [46]. The authors stated that despite of the large area of the London LEZ, the predicted changes in NO_2_ (and PM_10_) were generally small. Their modelled results stay partly in contrast to the prognoses published by Tonne et al. [79], who estimated for the London Congesting Charge Scheme a small decrease for NO_2_ of −0.64 µg/m^3^ only, corresponding to −1.1%.

The INTARESE project [80] modeled NO_2_ concentration changes for both LEZs in Rome and confirmed this finding of only small additional gains by stricter traffic restrictions. The main reductions were expected to be achieved already by excluding Euro 0 cars: −2.3 µg/m^3^ or −3.0 µg/m^3^. If only Euro 4 cars were allowed to enter the LEZs the reductions were expected to increase only slightly to −3.0 µg/m^3^ or −4.1 µg/m^3^ [81].

The “Stockholm Trial” involved a road pricing system to improve the air quality and reduce traffic congestion. The test period of the trial was January 3, 2006 to July 31, 2006. Vehicles travelling into and out of the charge cordon were charged for every passage during weekdays. Annual mean contributions to total levels of nitrogen oxides from emissions from road traffic with and without charges according to the Stockholm Trial were estimated. NO_x_ concentrations were lowered in periods with charges, but the study showed a small decrease only: −0.23 µg/m^3^ (Greater Stockholm) and −0.81 µg/m^3^ (inner city) [82]. No multivariable modeling was tried.

Boogaard et al. [83] analyzed measurements of NO_2_ and NO_x_ conducted simultaneously at eight streets, six urban background locations and four suburban background locations before (2008) and two years after implementation of an LEZ (2010) in five cities of The Netherlands (8 index stations, 4 reference stations). Index concentrations were lower in 2010 than in 2008 (NO_2_: −4.5 µg/m^3^, NO_x_: −6.1 µg/m^3^) but the differences were not statistically different. The study performed only crude comparisons and did not apply regression techniques to adjust for covariables.

The present study can be regarded as one of the most comprehensive approaches so far, analysing measurement data of nitrogen oxides concentrations in order to assess LEZ effects. The LEZ pollutant group 1 reduction effect on nitrogen oxides (NO_2_, NO, and NO_x_) was estimated as being no higher than 2 µg/m^3^ at all index stations and index traffic stations, i.e., no higher than about 4%. This estimate based on measurement data can be rated as the most profound currently available. This result also needs to be interpreted in the light of the existing EU limit values because LEZs are often supposed to be the most effective measure that cities can take to reduce air pollution problems in their area [84]. The respective NO_2_ concentration limit [24] enforced in Germany since 2010 is 40 µg/m^3^ (1 year average). Values are in excess and about 69% of all German traffic stations showed annual averages higher than 40 µg/m^3^ [25]. The four week averages of NO_2_ concentrations at index stations after LEZ introduction were found to be 55 µg/m^3^ (median and mean) or 82 µg/m^3^ (95th percentile). It follows that the estimated reduction of NO_2_ concentrations in the range of 2 µg/m^3^ appears to be of negligible impact when the current concentration levels should be lowered to the EU limit. The same judgement seems to apply on the EU level where the NO_2_ concentrations were reported to show a pronounced excess in many cities [26].

Regarding the information from the HBEFA [85] for real driving conditions in Germany, Austria and Switzerland with respect to vehicles that meet Euro 5 and 6 emission standards, no noteworthy reductions of NO_2_ and NO_x_ immissions are to be expected until a remarkable share of vehicles with NO_x_ after treatment systems (Euro 5 for HD trucks and Euro 6 for passenger cars) will be on the street [85].

The Handbook of Emission Factors for Road Transport (HBEFA) was originally developed on behalf of the Environmental Protection Agencies of Germany, Switzerland and Austria. In the meantime, further countries (Sweden, Norway, France) as well as the JRC (European Research Center of the European Commission) are supporting HBEFA. HBEFA provides emission factors, i.e. the specific emission in g/km for all current vehicle categories (PC, LDV, HDV, buses and motor cycles), each divided into different categories, for a wide variety of traffic situations (http://www.hbefa.net/e/index.html).

Interestingly, remarkable differences in NO_x_ and NO_2_ emissions from passenger cars and light duty vehicles are documented when low test cycle emissions were compared with relatively higher NO_x_/NO_2_ concentrations measured along roadsides [86], [87].

We analysed PM_10_ concentrations additionally [32] from 19 German LEZs. From about 2005 until the end of 2009 continuous half-hour measurement values as well as gravimetrically determined daily measurements of PM_10_ were collected. Two continuous procedures were used to measure mean PM_10_ concentrations per half-hour intervals [38], [88]:

Absorption of β-radiation (BA). The particulate matter is deposited on a filter tape and the change in β-ray transmission is measured.
Tapered Element Oscillating Microbalance (TEOM). An inertial balance directly measures the mass collected on an exchangeable filter cartridge by monitoring the corresponding frequency changes of a tapered element.

In addition, gravimetric samplers were used to measure daily averages of PM_10_ concentrations [49], [88], [89]. 2,110,803 quadruplets of continuous PM_10_ and 15,735 gravimetric quadruples were identified leading to 61,169 quadruplets based on daily PM_10_ averages. The analyses showed that best LEZ effect estimates were ≤0.2 µg/m^3^ at all index stations, i.e., the relative PM_10_ reduction ≤1%. Best estimates at all index stations near traffic (excluding urban background and industry index stations) were below 1 µg/m^3^ (less than 5%, resp). Effects were smaller than predicted prior to the introduction of LEZs. Limited data (1750 quadruplets of monthly averages) were also available to estimate the effects on soot parameters (elemental carbon, organic carbon and total carbon). The average of total carbon concentrations was estimated as 13 µg/m^3^ and LEZ effect estimates were about −0.55 µg/m^3^ or −4.2%. For PM_2.5_ only 650 quadruplets based on half-hour data and 99 quadruplets of daily concentration averages could be analyzed. The PM_2.5_ concentration mean was found at 17 µg/m^3^. All LEZ effect estimates on PM_2.5_ were positive, i.e., no indication of reduced concentrations after the introduction of the LEZs was found.

Due to the proven marginal reduction of nitrogen oxide concentrations (NO, NO_2_, NO_x_), LEZ as a regulatory action cannot be seen as an efficient measure to substantially reduce ambient nitrogen oxide exposures in the cities. Beyond that, this result is in good accordance to the effectiveness of LEZs on the reduction of PM_10_, too [32]. As predicted [33], long-term compliance problems with ambient air NO_2_ concentrations should be expected even if LEZs were introduced or enlarged for the purpose of NO_2_ reductions in cities.

The approach can be extended to account for other variables that are considered relevant [45]. Such data can only be used if these data are homogeneously available at all index and reference stations and are also available before and after the introduction of LEZs. Traffic density and car fleet properties are such variables of interest that do not meet the inclusion criteria: there are almost no data available in Germany to describe differences in flow of traffic and car fleet properties between index and reference stations and across time. To put this into perspective, we like to note first that changes in traffic density and car fleet properties are potentially affected by LEZs. It follows that traffic density and fleet properties should be considered as potential outcomes of LEZ introduction and not only as confounders of LEZ effects. This means that these data must not be accounted for by covariables in regression modelling even if the data were available in such a way that the inclusion criteria were met. Anyhow, authors who described changes in traffic-flow in Berlin argued against the interpretation that LEZs caused such displacements of traffic-flow from inside the LEZ to the reference stations [31]. Second, we note that the missing information on traffic density and fleet properties can be used to argue for biases in both directions. On the ones side, traffic could be displaced from the LEZ area to the reference stations outside so that the concentrations are underestimated inside but overestimated outside the LEZ, causing a potential overestimate of the LEZ effect. On the other side, if the car fleet is renewed not only inside the LEZ but also outside at the reference stations this may lead to a potential underestimate of the LEZ effect. We cannot conclude, therefore, on the direction of the potential bias.

The data analyzed in this study are the only available longitudinal measuring data to investigate the development of nitrogen oxide concentrations before and after the introduction of LEZs in Germany. We conclude that the material used can be considered as “data best available”. Interpretations are limited, however, because spatial representativeness of the measuring sites can be disputed. It is unknown whether these data can be used to reliably estimate the exposures of citizens living in the LEZs. Since this is not only a problem of German measuring networks but an issue on the European level a research project was started to investigate the representativeness of measurement sites [78]. The authors concluded that measurements at the background stations are of greater importance than the data collected at the hot spots (traffic stations). Other limitations of hot spot data result from the fact that the citizens living in the LEZ area spend most of their time indoors and that indoor pollution data differ from hot spot outdoor concentrations [90], [91].

## Conclusions

This is the first comprehensive approach to assess effects of LEZs on NO_2_, NO and NO_x_ concentrations with the help of measurement data on the Federal level in Germany. Reductions due to introducing LEZs of pollutant group 1 were estimated to be limited by 2 µg/m^3^ (or 4%). The 4-week averages of NO_2_ concentrations at index stations after LEZ introduction were found to be 55 µg/m^3^ (median and mean) or 82 µg/m^3^ (95th percentile). The NO_2_ concentration limit [24] enforced in Germany since 2010 is 40 µg/m^3^ (1 year average). Concerning the expenditure of regulations and controls which are required to introduce and operate LEZs in cities, the proven impact of LEZs on the reduction of NO_2_ ambient air concentrations with at a maximum of 4% in the first phase is very small.

## Supporting Information

File S1 **Contains Tables S1–S4 and Figures S1–S19.** **Table S1:** Detailed results on NO_2_ - quadruplet analyses by linear (additive) log-linear (multiplicative) regression models. **Table S2:** NO: Log-linear (multiplicative) model 1 evaluating the quadruplets of pooled continuous and diffuse sampler NO-measurements. Regression coefficient, robust standard errors of coefficient, t-statistic, two-sided P-value, and 95%-confidence interval of coefficient. The relative LEZ effect estimate is given by the coefficient E (<1: concentration is lowered by LEZ). **Table S3:** NO_x_: Quadruplets of pooled continuous and diffuse sampler NO_x_-measurements: index stations (Ind), reference stations (Ref) before (pre) and after (post) introduction of LEZ. Ind.diff and Ref.diff denote differences between index measurements and between reference measurements (negative post-pre differences indicate lower values after introduction of LEZ). **Table S4:** NO_x_: Linear (additive) model 1 evaluating the quadruplets of pooled continuous and diffuse sampler NO_x_-measurements. Regression coefficient, robust standard errors of coefficient, t-statistic, two-sided P-value, and 95%-confidence interval of coefficient. The absolute LEZ effect estimate is given by the coefficient E in µg/m^3^ (<0: concentration is lowered by LEZ). **Table S5:** NO_x_: Log-linear (multiplicative) model 1 evaluating the quadruplets of pooled continuous and diffuse sampler NO_x_-measurements. Regression coefficient, robust standard errors of coefficient, t-statistic, two-sided P-value, and 95%-confidence interval of coefficient. The relative LEZ effect estimate is given by the coefficient E (<1: concentration is lowered by LEZ). **Figure S1:** Low emission zone **Herrenberg** (marked area), implemented in 2009-01-01 (modified from www.map24.de). One index station: 1)DEBW135 Hindenburger Straße, no NO, no NO_x_. One reference station outside the low emission zone: 2)DEBW112 Gärtringen (not included in the figure since located approx. 5 km north of low emission zone). **Figure S2:** Low emission zone **Ilsfeld** (marked area), implemented in 2008-03-01 (modified from www.map24.de). One index station: 1)DEBW133 König-Wilhelm-Straße, no NO, no NO_x_. One reference station outside the low emission zone: 2)DEBW034 Waiblingen (not included in the figure since located approx. 24 km south of low emission zone). **Figure S3:** Low emission zone **Karlsruhe** (marked area), implemented in 2009-01-01 (modified from www.map24.de). One index station: 1)DEBW126 Kriegsstraße, no NO_2_, no NO_x_. Two reference stations outside the low emission zone: 2)DEBW001 Karlsruhe-Mitte 3)DEBW004 Eggenstein (not included in the figure since located approx. 6 km north of low emission zone). **Figure S4:** Low emission zone **Ludwigsburg** (marked area), implemented in 2008-03-01 (modified from www.map24.de). Two index stations: 1)DEBW024 Weimar-/Schweizerstraße 2)DEBW017 Friedrichstraße. One reference station outside the low emission zone: 3)DEBW034 Waiblingen (not included in the figure since located approx. 7 km south east of low emission zone). **Figure S5:** Low emission zone **Mannheim** (marked area), implemented in 2008-03-01 (modified from www.map24.de). Two index stations: 1)DEBW006 Mannheim-Mitte 2)DEBW098 Friedrichsring U2. Two reference stations outside the low emission zone: 3)DEBW005 Mannheim Nord (not included in the figure since located approx. 4 km north of low emission zone) 4)DEBW007 Mannheim-Süd (not included in the figure since located approx. 5 km south of low emission zone). **Figure S6:** Low emission zone **Reutlingen** (marked area), implemented in 2008-03-01 (modified from www.map24.de). One index station: 1)DEBW027 Ebertstraße. Two reference stations outside the low emission zone: 2)DEBW042 Bernhausen (not included in the figure since located approx. 20 km north of low emission zone) 3)DEBW117 Gärtringen (not included in the figure since located approx. 28 km west of low emission zone). **Figure S7:** Low emission zone **Stuttgart** (marked area), implemented in 2008-03-01 (modified from www.map24.de). Six index stations: 1)DEBW011 Zuffenhausen 2)DEBW013 Seuberstraße 3)DEBW099 Arnulf-Klett-Platz 4)DEBW116 Hohenheimer Straße 5)DEBW118 Am Neckartor 6)DEBW134 Waiblinger Straße. Two reference stations outside the low emission zone: 7)DEBW034 Waiblingen 8)DEBW042 Bernhausen (not included in the figure since located approx. 2 km south of low emission zone). **Figure S8:** Low emission zone **Tübingen** (marked area), implemented in 2008-03-01 (modified from www.map24.de). One index station: 1)DEBW107 Derendingerstraße. One reference station outside the low emission zone: 2)DEBW112 Gärtringen (not included in the figure since located approx. 15 km north west of low emission zone). **Figure S9:** Low emission zone **Augsburg** (marked area), implemented in 2009-07-01 (modified from www.map24.de). Three index stations: 1)DEBY007 Bourges-Platz 2)DEBY110 Karlstraße 3)DEBY006 Königsplatz. One reference station outside the low emission zone: 4)DEBY099 LfU (not included in the figure since located approx. 3 km south of low emission zone). **Figure S10:** Low emission zone **Munich** (marked area), implemented in 2008-10-01 (modified from www.map24.de). Five index stations: 1)DEBY037 Stachus 2)DEBY039 Lothstraße 3)DEBY085 Luise-Kiesselbach-Platz 4)DEBY114 Prinzregentenstraße 5)DEBY115 Landshuter Allee. Three reference stations outside the low emission zone: 6)DEBY043 Moosach, no PM_10_ 7)DEBY089Johanneskirchen 8)DEBY109 Andechs/Rothenfeld (not included in the figure since located approx. 27 km south west of low emission zone). **Figure S11a:** Low emission zone **Berlin Blume-Messnetz** (marked area), implemented in 2008-01-01 (modified from www.map24.de).Five index stations: 1)DEBE018 B Schöneberg-Belziger Straße 2)DEBE034 B Neukölln-Nansenstraße 3)DEBE064 B Neukölln-Karl-Marx-Straße 76 4)DEBE065 B Friedrichshain-Frankfurter Allee 5)DEBE067 B Hardenbergplatz. Nine reference stations outside the low emission zone: 6)DEBE061 B Steglitz-Schildhornstraße 7)DEBE062 B Frohnau, Funkturm (not included in the figure since located approx. 13 km north of low emission zone) 8)DEBE063 B Neukölln-Silbersteinstraße) 9)DEBE066 B Karlshorst-Rheingoldstraße, no PM_10_ (not included in the figure since located approx. 5 km east of low emission zone) 10)DEBE010 B Wedding-Amrumer Straße 11)DEBE027 B Marienfelde-Schichauweg (not included in the figure since located approx. 8 km south of low emission zone) 12)DEBE032 B Grunewald (not included in the figure since located approx. 4 km south west of low emission zone) 13)DEBE051 B Buch (not included in the figure since located approx. 12 km north east of low emission zone) 14)DEBE056 B Friedrichshagen (not included in the figure since located approx. 14 km south east of low emission zone). **Figure S11b:** Low emission zone **Berlin RUBIS-Messnetz** (marked area), implemented in 2008-01-01 (modified from www.map24.de). Ten index stations: 1)DEBE530 Hauptstraße 30 2)DEBE504 Beusselstraße 66 3)DEBE537 Alt Moabit 63 4)DEBE545 Sonnenallee 68 5)DEBE547 Landsberger Allee 6–8 6)DEBE517 Neukölln-Nansenstraße 7)DEBE519 Friedrichshain-Frankfurter Allee 8)DEBE555 Herrmannplatz Laterne 21 9)DEBE562 Friedrichstraße Laterne 156 10)DEBE525 Leipziger Straße 32. Twelve reference stations outside the low emission zone:11) DEBE501 Berliner Allee 118 12)DEBE577 Buch, no NO, no NO_x_ (not included in the figure since located approx. 12 km north of low emission zone) 13)DEBE507 Grünauer Straße 4 (not included in the figure since located approx. 9 km south east of low emission zone) 14)DEBE539 Schloßstraße 29 15)DEBE542 Tempelhofer Damm 148 16)DEBE513 Spreestraße 2 (not included in the figure since located approx. 5 km south east of low emission zone) 17)DEBE514 Alt Friedrichsfelde 8a (not included in the figure since located approx. 3 km east of low emission zone) 18)DEBE521 Steglitz-Schildhornstraße 19)DEBE559 Buschkrugallee Laterne 3 20)DEBE522 Neukölln-Silbersteinstraße1 21)DEBE573 Badstraße 22)DEBE576 Spandau, Klosterstraße 12 (not included in the figure since located approx. 6 km west of low emission zone). **Figure S12:** Low emission zone **Frankfurt a.M.** (marked area), implemented in 2008-10-01 (modified from www.map24.de). One index station: 1)DEHE041 Frankfurt-Friedb.Ldstr. Three reference stations outside the low emission zone: 2)DEHE008 Frankfurt-Ost 3)DEHE011 Hanau (not included in the figure since located approx. 13 km east of low emission zone) 4)DEHE005 Frankfurt-Höchst. **Figure S13:** Low emission zone **Hannover** (marked area), implemented in 2008-01-01 (modified from www.map24.de). One index station: 1)DENI048 Hannover Verkehr. Four reference stations outside the low emission zone: 2)DENI054 Hannover 3)DENI011 Braunschweig, Broizemer Steinberg (not included in the figure since located approx. 49 km east of low emission zone) 4)DENI041 Weserbergland/Rinteln, Brugfeldsweide (not included in the figure since located approx. 48 km south west of low emission zone) 5)DENI052 Allertal/Walsrode, Auf dem Kamp 8 (not included in the figure since located approx. 47 km north of low emission zone). **Figure S14:** Low emission zone **Dortmund** (marked area), implemented in 2008-10-01, but Brackelerstr. 2008-01-01 (modified from www.map24.de). Four index stations: 1)DENW101 Steinstraße 2)DENW136 Brackeler Straße 3)DENW184 Westfalendamm 190, no NO, no NO_x_, no PM_10_ 4)DENW185 Rheinlanddamm 5–7, no NO, no NO_x_, no PM_10_. Four reference stations outside the low emission zone: 5)DENW002 Datteln-Hagem (not included in the figure since located approx. 15 km north west of low emission zone) 6)DENW008 Do-Eving 7)DENW029 Hattingen, An der Becke (not included in the figure since located approx. 19 km south west of low emission zone) 8)DENW179 Schwerte (not included in the figure since located approx. 8 km south of low emission zone). **Figure S15:** Low emission zone **Duisburg** (marked area), implemented in 2008-10-01 (modified from www.map24.de).Three index stations: 1)DENW034 Duisburg-Walsum 2)DENW040 Duisburg-Buchholz 3)DENW112 Kardinal-Galen-Straße. One reference station outside the low emission zone: 4)DENW038 45476 Mühlheim, Neustadtstraße (not included in the figure since located approx. 5 km east of low emission zone). **Figure S16:** Low emission zone **Düsseldorf** (marked area), implemented in 2009-02-15 (modified from www.map24.de). Two index stations: 1)DENW082 Corneliusstraße 2)DENW216 Düsseldorf-Bilk, no NO, no NO_x_, no PM_10_. Four reference stations outside the low emission zone: 3)DENW042 Krefeld-Linn (not included in the figure since located approx. 14 km north west of low emission zone) 4)DENW071 Düsseldorf-Lörick (not included in the figure since located approx. 3 km west of low emission zone) 5)DENW078 Ratingen-Tiefenbroich (not included in the figure since located approx. 6 km north east of low emission zone) 6)DENW116 Krefeld Hafen (not included in the figure since located approx. 12 km north west of low emission zone). **Figure S17:** Low emission zone **Essen** (marked area), implemented in 2008-10-01 (modified from www.map24.de). Eight index stations: 1)DENW043Ost Steeler Straße 2)DENW134 Gladbecker Straße 3)DENW135 Hombrucher Straße 4)DENW161 Alfredstraße 9/11, no NO, no NO_x_, no PM_10_ 5)DENW168 Gladbecker Straße 245, no NO, no NO_x_, no PM_10_ 6)DENW169 In der Baumschule 7, no NO, no NO_x_, no PM_10_ 7)DENW171 Hombrucherstraße 21/23, no NO, no NO_x_, no PM_10_ 8)DENW215 Hausackerstraße 11, no NO, no NO_x_, no PM_10_. Three reference stations outside the low emission zone: 9)DENW024 Essen-Vogelheim 10)DENW029 Hattingen-Blankenstein (not included in the figure since located approx. 10 km south east of low emission zone), 11) DENW162 Brückstraße 29, no NO, no NO_x_, no PM_10_ (not included in the figure since located approx. 4 km south of low emission zone). **Figure S18:** Low emission zone **Cologne** (marked area), implemented in 2008-01-01 (modified from www.map24.de). Seven index stations: 1)DENW148 Justinianstraße 13–15, no NO, no NO_x_, no PM_10_ 2)DENW151 Neumarkt 25, no NO, no NO_x_, no PM_10_ 3)DENW153 Tunisstraße/Elstergasse, no NO, no NO_x_, no PM_10_ 4)DENW164 Hohenstaufenring 57A, no NO, no NO_x_, no PM_10_ 5)DENW198 Gereonsdriesch 21, no NO, no NO_x_, no PM_10_ 6)DENW211 Clevischer Ring 3 7)DENW212 Turiner Straße. Four reference stations outside the low emission zone: 8)DENW053 Cologne-Chorweiler (not included in the figure since located approx. 9 km north west of low emission zone) 9)DENW058 Hürth (not included in the figure since located approx. 7 km south west of low emission zone), 10)DENW059 Cologne-Rodenkirchen (not included in the figure since located approx. 4 km south of low emission zone), 11)DENW079 Leverkusen-Manfort (not included in the figure since located approx. 7 km north of low emission zone). **Figure S19:** Low emission zone **Wuppertal** (marked area), implemented in 2009-02-15 (modified from www.map24.de). Two index stations: 1)DENW114 Wuppertal-Langerfeld, no NO, no NO_x_ 2)DENW189 Wuppertal Gathe. Two reference stations outside the low emission zone: 3)DENW029 Hattingen-Blankenstein (not included in the figure since located approx. 13 km north of low emission zone) 4)DENW080 Solingen-Wald (not included in the figure since located approx. 5 km south west of low emission zone).
(DOCX)
