# Supplementary material for: Effectiveness of Low Emission Zones: Large Scale Analysis of Changes in Environmental NO2, NO and NOx Concentrations in 17 German Cities
Source: PLoS One. 2014 Aug 12;9(8):e102999. doi: 10.1371/journal.pone.0102999 (PMC4130490; doi:10.1371/journal.pone.0102999)
Supplement: File S1 — Contains Tables S1–S4 and Figures S1–S19. Table S1: Detailed results on NO2 - quadruplet analyses by linear (additive) log-linear (multiplicative) regression models. Table S2: NO: Log-linear (multiplicative) model 1 evaluating the quadruplets of pooled continuous and diffuse sampler NO-measurements. Regression coefficient, robust standard errors of coefficient, t-statistic, two-sided P-value, and 95%-confidence interval of coefficient. The relative LEZ effect estimate is given by the coefficient E (<1: concentration is lowered by LEZ). Table S3: NOx: Quadruplets of pooled continuous and diffuse sampler NOx-measurements: index stations (Ind), reference stations (Ref) before (pre) and after (post) introduction of LEZ. Ind.diff and Ref.diff denote differences between index measurements and between reference measurements (negative post-pre differences indicate lower values after introduction of LEZ). Table S4: NOx: Linear (additive) model 1 evaluating the quadruplets of pooled continuous and diffuse sampler NOx-measurements. Regression coefficient, robust standard errors of coefficient, t-statistic, two-sided P-value, and 95%-confidence interval of coefficient. The absolute LEZ effect estimate is given by the coefficient E in µg/m3 (<0: concentration is lowered by LEZ). Table S5: NOx: Log-linear (multiplicative) model 1 evaluating the quadruplets of pooled continuous and diffuse sampler NOx-measurements. Regression coefficient, robust standard errors of coefficient, t-statistic, two-sided P-value, and 95%-confidence interval of coefficient. The relative LEZ effect estimate is given by the coefficient E (<1: concentration is lowered by LEZ). Figure S1: Low emission zone Herrenberg (marked area), implemented in 2009-01-01 (modified from www.map24.de). One index station: 1)DEBW135 Hindenburger Straße, no NO, no NOx. One reference station outside the low emission zone: 2)DEBW112 Gärtringen (not included in the figure since located approx. 5 km north of low emission zone). F [file pone.0102999.s001.docx]

**Table S1:** Detailed results on NO_2_ - quadruplet analyses by linear (additive) log-linear (multiplicative) regression models.

| Evaluation  Period, | Type  of Measurement, | Model  Type | Number of  Federal States | Number of  LEZs | Number  of  Quadruplets | E in μg/m3 (additive) | Two-sided  P value (additive) | 95%  confidence  interval (additive) | | (E-1) *100%  (multipl) | Two-sided  P value (multipl) | 95%  confidence  interval (multipl) | |
| --- | --- | --- | --- | --- | --- | --- | --- | --- | --- | --- | --- | --- | --- |
| 0.5h | Continuous | 0 | 6 | 17 | 3038781 | -0.968 | <0.001 | -0.999 | -0.938 | -2.165 | <0.001 | -2226 | -2.104 |
| 0.5h | Continuous | 1 | 6 | 17 | 3038781 | -1.112 | <0.001 | -1.137 | -1.087 | -2.093 | <0.001 | -2.146 | -2.04 |
| 0.5h | Continuous | 2 | 6 | 17 | 3038781 | -1.85 | <0.001 | -1.881 | -1.819 | -3.922 | <0.001 | -4.25 | -3.592 |
| 4 weeks | Continuous | 0 | 6 | 17 | 2277 | -1.05 | <0.001 | -1.343 | 0.756 | -2.293 | <0.001 | -2.837 | -1.746 |
| 4 weeks | Continuous | 1 | 6 | 17 | 2277 | -0.785 | <0.001 | -1.084 | -0.486 | -2.079 | <0.001 | -2.705 | -1.448 |
| 4 weeks | Continuous | 2 | 6 | 17 | 2277 | -1.983 | <0.001 | -2.39 | -1.575 | -4.891 | 0.014 | -8.608 | -1.023 |
| 4 weeks | Diffuse | 0 | 2 | 2 | 1818 | -1.599 | <0.001 | -1.951 | -1.248 | -2.813 | <0.001 | -3.402 | -2.22 |
| 4 weeks | Diffuse | 1 | 2 | 2 | 1818 | -1.182 | <0.001 | -1.55 | -0.814 | -1.841 | <0.001 | -2.52 | -1.158 |
| 4 weeks | Diffuse | 2 | 2 | 2 | 1818 | -2.81 | <0.001 | -2.544 | -1.618 | -4.778 | 0.019 | -8.608 | -0.788 |
| 4 weeks | Total | 0 | 6 | 17 | 4095 | -1.017 | <0.001 | -1.249 | -0.786 | -2.491 | <0.001 | -2.888 | -2.092 |
| 4 weeks | Total | 1 | 6 | 17 | 4095 | -0.826 | <0.001 | -1.068 | -0.585 | -1.971 | <0.001 | -2.459 | -1.48 |
| 4 weeks | Total | 2 | 6 | 17 | 4095 | -1.732 | <0.001 | -2.032 | -1.433 | -3.878 | 0.018 | -6.98 | -0.672 |

Model 0 covariables: difference in reference stations Ref.diff in μg/m^3^

Model 1 covariables: Model 0 covariable + centered reference baseline concentration Ref.base in μg/m^3^, centered index baseline concentration Ind.base in μg/m^3^, Diff 1/H = difference in 1/(height of inversion layer) in 1/m, Diff 1/V = 1/(wind velocity +0.01) in (m/s)^-1^, difference in 1/P = 1/(amount of precipitation +0.01) (mm/h) ^-1^, centered difference in time Time.diff in years.

Model 2 covariables: Model 1 covariables + period of school holidays (yes/no), period of environmental bonus paid (yes/no), periods when trucks were not allowed to enter the areas (yes/no)

**Table S2:** NO: Log-linear (multiplicative) model 1 evaluating the quadruplets of pooled continuous and diffuse sampler NO-measurements. Regression coefficient, robust standard errors of coefficient, t-statistic, two-sided P-value, and 95%-confidence interval of coefficient. The relative LEZ effect estimate is given by the coefficient E (< 1: concentration is lowered by LEZ).

| ln Ind.diff | Coef. | Std. Err. | t | p | 95% Conf. | Interval |
| --- | --- | --- | --- | --- | --- | --- |
| ln Ref.diff | 0.461 | 0.018 | 25.4 | <0.001 | 0.426 | 0.497 |
| ln Ref.base | 0.003 | 0.005 | 0.65 | 0.516 | -0.007 | 0.013 |
| ln Ind.base | -0.049 | 0.012 | -4.16 | <0.001 | -0.072 | -0.026 |
| Diff ln 1/H | -0.022 | 0.006 | -3.99 | <0.001 | -0.033 | -0.011 |
| Diff ln 1/V | 0.036 | 0.006 | 6.14 | <0.001 | 0.024 | 0.047 |
| Diff ln 1/P | 0.005 | 0.006 | 0.90 | 0.367 | -0.006 | 0.017 |
| ln Time.diff | 0.009 | 0.010 | 0.88 | 0.378 | -0.011 | 0.029 |
| ln E | -0.032 | 0.005 | -6.89 | <0.001 | -0.041 | -0.023 |

relative effect E = 0 .968, 95% Conf. Interval = 0 .959, 0.977

Covariables, before taking logs: difference in reference stations Ref.diff in μg/m^3^, centered reference baseline concentration Ref.base in μg/m^3^, centered index baseline concentration Ind.base in μg/m^3^, 1/H = 1/(height of inversion layer) in 1/m, 1/V = 1/(wind velocity +0.01) in (m/s)^-1^, 1/ P = 1/(amount of precipitation +0.01) (mm/h) ^-1^, centered difference in time Time.diff in years.

**Table S3:** NO_x_: Quadruplets of pooled continuous and diffuse sampler NO_x_-measurements: index stations (Ind), reference stations (Ref) before (pre) and after (post) introduction of LEZ. Ind.diff and Ref.diff denote differences between index measurements and between reference measurements (negative post-pre differences indicate lower values after introduction of LEZ).

| Statistic | Ind,pre | Ind,post | Ref,pre | Ref,post | Ind.diff. | Ref.diff |
| --- | --- | --- | --- | --- | --- | --- |
| N | 4005 | 4005 | 4005 | 4005 | 4005 | 4005 |
| min | 16 | 14 | 8.0 | 7.2 | -118 | -70 |
| p5 | 30 | 29 | 14 | 12 | -32 | -32 |
| p50 | 107.4 | 100.4 | 77.1 | 68.3 | -5.63 | -4.30 |
| mean | 105.929 | 100.662 | 74.063 | 68.968 | -5.267 | -5.095 |
| p95 | 171 | 170 | 149 | 141 | 28 | 19 |
| max | 345 | 387 | 214 | 176 | 108 | 80 |

N: number of quadruplets, Min: minimum, p5: 5^th^ percentile, p50: median,

mean: arithmetic average, p95: 95^th^ percentile, max: maximum

**Table S4:** NO_x_: Linear (additive) model 1 evaluating the quadruplets of pooled continuous and diffuse sampler NO_x_-measurements. Regression coefficient, robust standard errors of coefficient, t-statistic, two-sided P-value, and 95%-confidence interval of coefficient. The absolute LEZ effect estimate is given by the coefficient E in μg/m^3^ (< 0: concentration is lowered by LEZ).

| Ind.diff | Coef. | Std. Err. | t | p | 95% Conf. | Interval |
| --- | --- | --- | --- | --- | --- | --- |
| Ref.diff | 0.710 | 0.020 | 36.0 | <0.001 | 0.671 | 0.748 |
| Ref.base | 0.649 | 0.026 | 25.4 | <0.001 | 0.599 | 0.700 |
| Ind.base | -0.688 | 0.021 | -32.3 | <0.001 | -0.73 | -0.646 |
| Diff 1/H | 56.5 | 44.3 | 1.28 | 0.202 | -30.4 | 143 |
| Diff 1/V | -0.928 | 0.33 | -2.81 | 0.005 | -1.576 | -0.280 |
| Diff 1/P | -0.071 | 0.068 | -1.04 | 0.297 | -0.205 | 0.062 |
| Time.diff | 0.699 | 0.428 | 1.63 | 0.103 | -0.141 | 1.538 |
| E | -1.74 | 0.303 | -5.74 | <0.001 | -2.334 | -1.145 |

Covariables: difference in reference stations Ref.diff in μg/m^3^, centered reference baseline concentration Ref.base in μg/m^3^, centered index baseline concentration Ind.base in μg/m^3^, Diff 1/H = difference in 1/(height of inversion layer) in 1/m, Diff 1/V = 1/(wind velocity +0.01) in (m/s)^-1^, difference in 1/P = 1/(amount of precipitation +0.01) (mm/h) ^-1^, centered difference in time Time.diff in years.

**Table S5** - NO_x_: Log-linear (multiplicative) model 1 evaluating the quadruplets of pooled continuous and diffuse sampler NO_x_-measurements. Regression coefficient, robust standard errors of coefficient, t-statistic, two-sided P-value, and 95%-confidence interval of coefficient. The relative LEZ effect estimate is given by the coefficient E (< 1: concentration is lowered by LEZ).

| ln Ind.diff | Coef. | Std. Err. | t | p | 95% Conf. | Interval |
| --- | --- | --- | --- | --- | --- | --- |
| ln Ref.diff | 0.461 | 0.017 | 26.4 | <0.001 | 0.427 | 0.495 |
| ln Ref.base | 0.005 | 0.003 | 1.64 | 0.102 | -0.001 | 0.012 |
| ln Ind.base | -0.053 | 0.015 | -3.41 | 0.001 | -0.083 | -0.023 |
| Diff ln 1/H | -0.013 | 0.004 | -3.59 | <0.001 | -0.021 | -0.006 |
| Diff ln 1/V | 0.018 | 0.004 | 4.27 | <0.001 | 0.010 | 0.026 |
| Diff ln 1/P | 0.005 | 0.004 | 1.33 | 0.185 | -0.002 | 0.012 |
| ln Time.diff | 0.007 | 0.007 | 1.02 | 0.308 | -0.007 | 0.021 |
| ln E | -0.024 | 0.003 | -7.71 | <0.001 | -0.031 | -0.018 |

relative effect E = 0 .976, 95% Conf. Interval = 0 .970, 0.982

Covariables, before taking logs: difference in reference stations Ref.diff in μg/m^3^, centered reference baseline concentration Ref.base in μg/m^3^, centered index baseline concentration Ind.base in μg/m^3^, 1/H = 1/(height of inversion layer) in 1/m, 1/V = 1/(wind velocity +0.01) in (m/s)^-1^, 1/ P = 1/(amount of precipitation +0.01) (mm/h) ^-1^, centered difference in time Time.diff in years.

In total, 19 cities with LEZs in 6 German Federal states could be identified and included into the study according to protocol. The LEZs Ilsfeld and Karlsruhe could only be analyzed for PM_10_ but not for nitrogen oxides. Thus, the PM_10_ study covered 19 LEZs but the nitrogen oxide study only 17 LEZs. Appendix B shows the names and locations of all index and reference stations and whether PM_10_, NO_2_, NO, and NO_x_ was available at the station for analysis. If we make no commentary all four concentrations were available. In all other cases we report which value is missing.

Baden-Württemberg

Herrenberg, Ilsfeld, Karlsruhe, Ludwigsburg, Mannheim, Reutlingen, Stuttgart, Tübingen

Bavaria

Augsburg, Munich

Berlin

Berlin

Hesse

Frankfurt a.M.

Lower Saxony

Hannover

North Rhine-Westphalia

Dortmund, Duisburg, Düsseldorf, Essen, Cologne, Wuppertal.

**Baden-Württemberg**


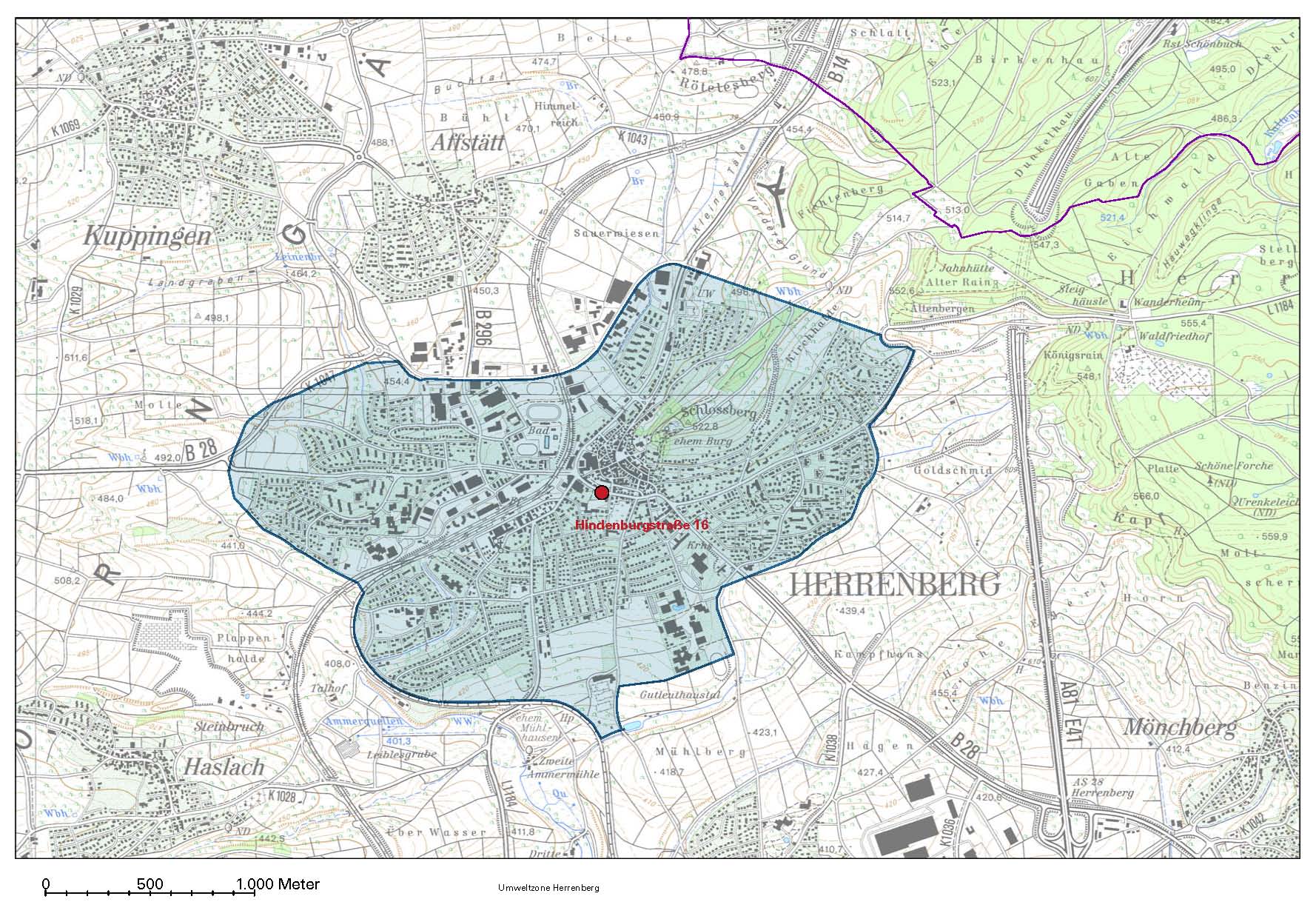


**➊**

**Figure S1:** Low emission zone **Herrenberg** (marked area), implemented in 2009-01-01 (modified from www.map24.de). One index station: ➊DEBW135 Hindenburger Straße, no NO, no NO_x_. One reference station outside the low emission zone: ➋DEBW112 Gärtringen (not included in the figure since located approx. 5 km north of low emission zone).


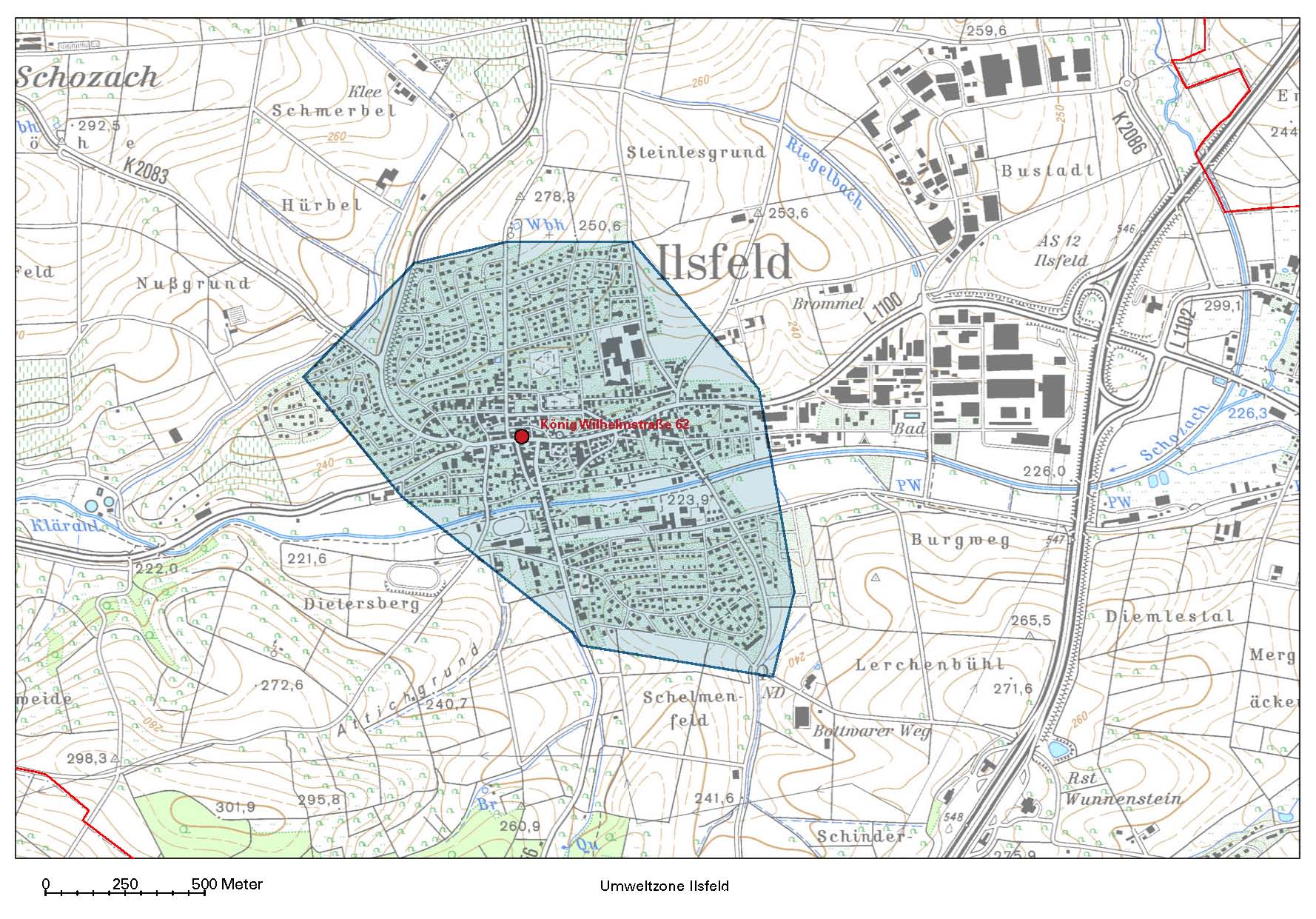


**➊**

**Figure S2:** Low emission zone **Ilsfeld** (marked area), implemented in 2008-03-01 (modified from www.map24.de). One index station: ➊DEBW133 König-Wilhelm-Straße, no NO, no NO_x_. One reference station outside the low emission zone: ➋DEBW034 Waiblingen (not included in the figure since located approx. 24 km south of low emission zone).


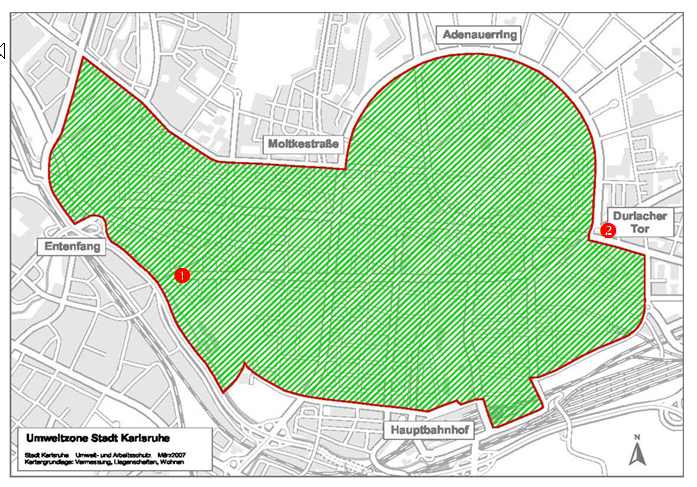


**Figure S3:** Low emission zone **Karlsruhe** (marked area), implemented in 2009-01-01 (modified from www.map24.de). One index station: ➊DEBW126 Kriegsstraße, no NO_2_, no NO_x_. Two reference stations outside the low emission zone: ➋DEBW001 Karlsruhe-Mitte ➌DEBW004 Eggenstein (not included in the figure since located approx. 6 km north of low emission zone).


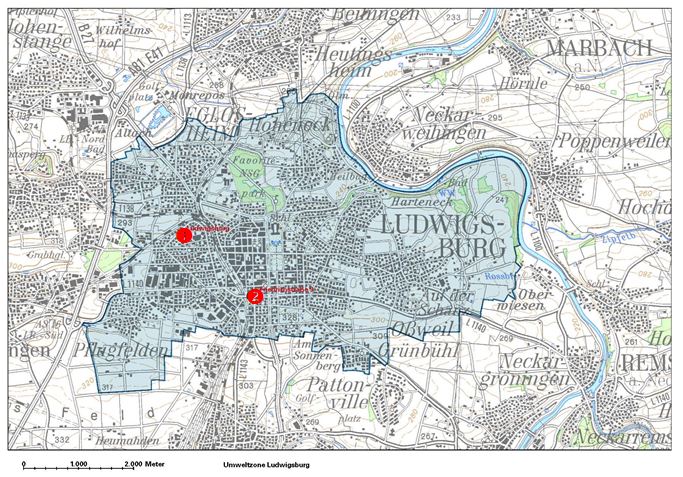


**Figure S4:** Low emission zone **Ludwigsburg** (marked area), implemented in

2008-03-01 (modified from www.map24.de). Two index stations: ➊DEBW024 Weimar-/Schweizerstraße ➋DEBW017 Friedrichstraße. One reference station outside the low emission zone: ➌DEBW034 Waiblingen (not included in the figure since located approx. 7 km south east of low emission zone).


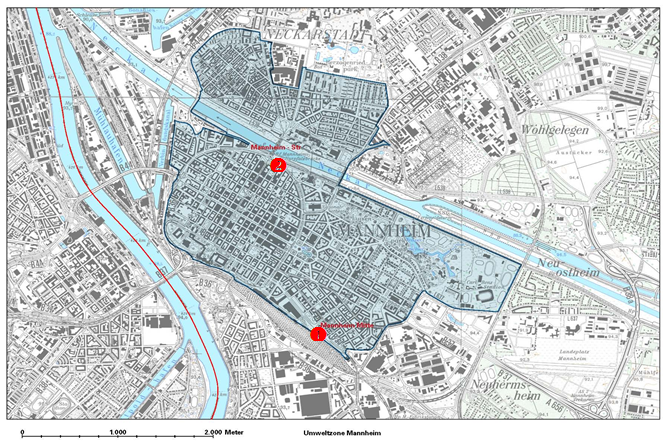


**Figure S5:** Low emission zone **Mannheim** (marked area), implemented in 2008-03-01 (modified from www.map24.de). Two index stations: ➊DEBW006 Mannheim-Mitte ➋DEBW098 Friedrichsring U2. Two reference stations outside the low emission zone: ➌DEBW005 Mannheim Nord (not included in the figure since located approx. 4 km north of low emission zone) ➍DEBW007 Mannheim-Süd (not included in the figure since located approx. 5 km south of low emission zone).


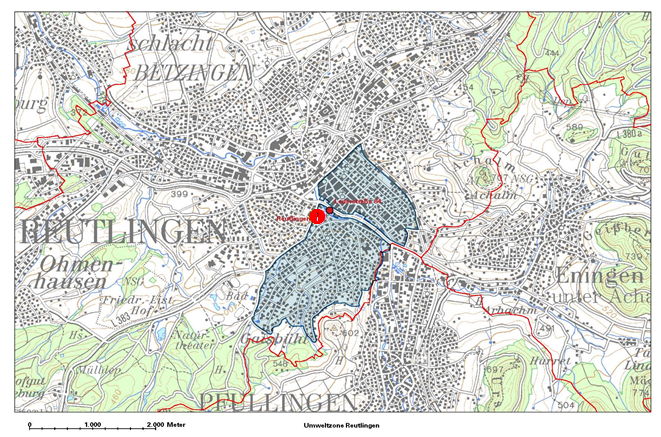


**Figure S6:** Low emission zone **Reutlingen** (marked area), implemented in 2008-03-01 (modified from www.map24.de). One index station: ➊DEBW027 Ebertstraße. Two reference stations outside the low emission zone: ➋DEBW042 Bernhausen (not included in the figure since located approx. 20 km north of low emission zone) ➌DEBW117 Gärtringen (not included in the figure since located approx. 28 km west of low emission zone).


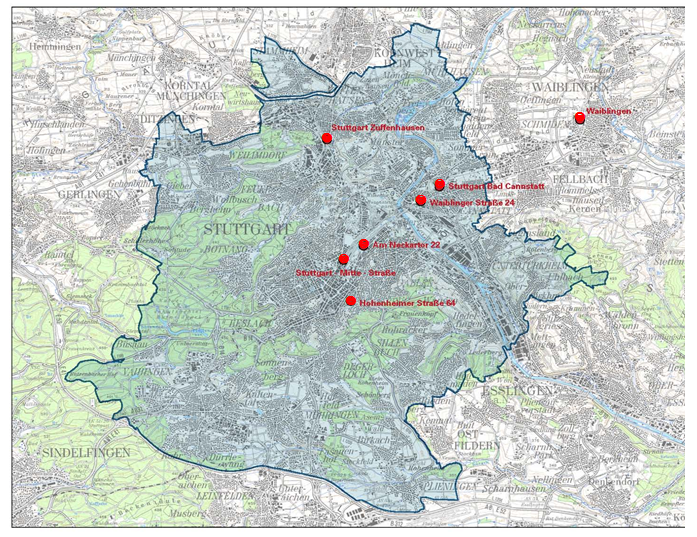


**Figure S7:** Low emission zone **Stuttgart** (marked area), implemented in 2008-03-01 (modified from www.map24.de). Six index stations: ➊DEBW011 Zuffenhausen ➋DEBW013 Seuberstraße ➌DEBW099 Arnulf-Klett-Platz ➍DEBW116 Hohenheimer Straße ➎DEBW118 Am Neckartor ➏DEBW134 Waiblinger Straße. Two reference stations outside the low emission zone: ➐DEBW034 Waiblingen ➑DEBW042 Bernhausen (not included in the figure since located approx. 2 km south of low emission zone).


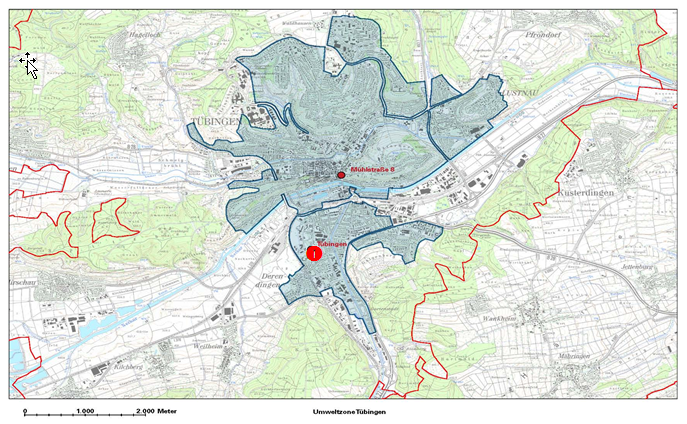


**Figure S8:** Low emission zone **Tübingen** (marked area), implemented in 2008-03-01 (modified from www.map24.de). One index station: ➊DEBW107 Derendingerstraße. One reference station outside the low emission zone: ➋DEBW112 Gärtringen (not included in the figure since located approx. 15 km north west of low emission zone)..

**Bavaria**


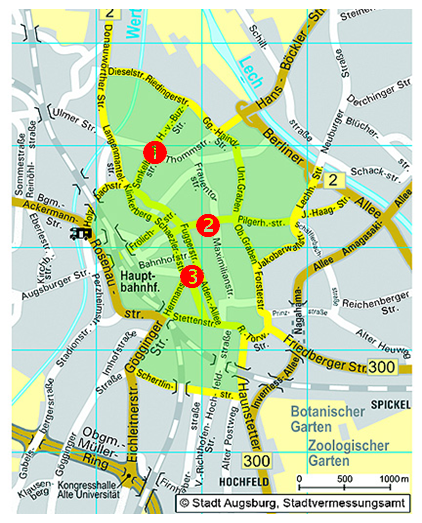


**Figure S9:** Low emission zone **Augsburg** (marked area), implemented in 2009-07-01 (modified from www.map24.de). Three index stations: ➊DEBY007 Bourges-Platz ➋DEBY110 Karlstraße ➌DEBY006 Königsplatz. One reference station outside the low emission zone: ➍ DEBY099 LfU (not included in the figure since located approx. 3 km south of low emission zone).


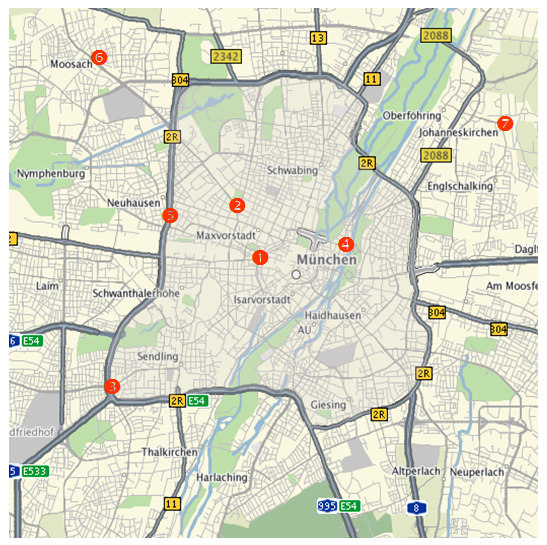


**Figure S10:** Low emission zone **Munich** (marked area), implemented in 2008-10-01 (modified from www.map24.de). Five index stations: ➊DEBY037 Stachus ➋DEBY039 Lothstraße ➌DEBY085 Luise-Kiesselbach-Platz ➍DEBY114 Prinzregentenstraße ➎DEBY115 Landshuter Allee. Three reference stations outside the low emission zone: ➏DEBY043 Moosach, no PM_10_  ➐DEBY089 Johanneskirchen ➑DEBY109 Andechs/Rothenfeld (not included in the figure since located approx. 27 km south west of low emission zone).

**Berlin**


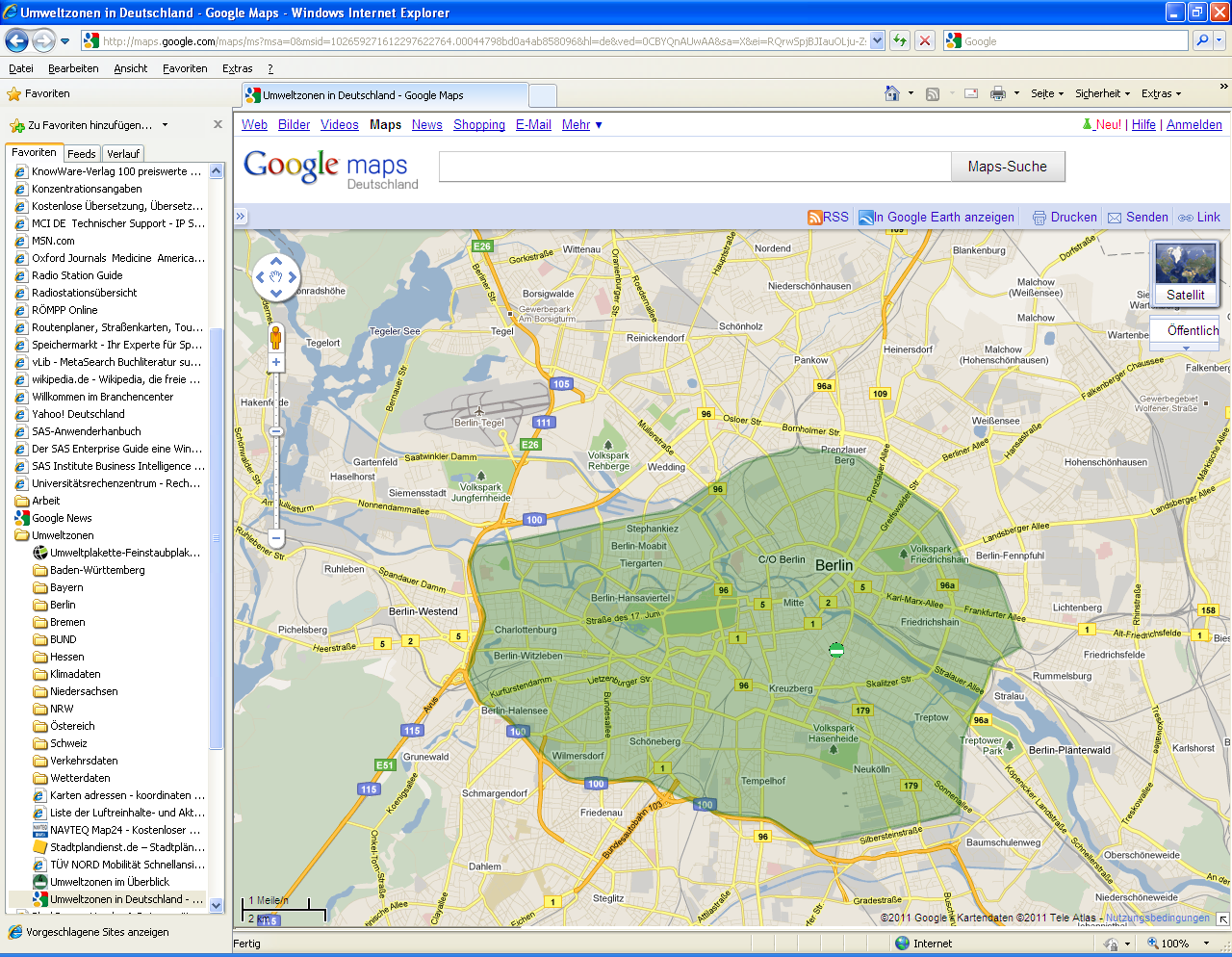


**➓**

**➏**

**➑**

**➊**

**➋**

**➌**

**➍**

**➎**

**Figure S11a:** Low emission zone **Berlin Blume-Messnetz** (marked area), implemented in 2008-01-01 (modified from www.map24.de).Five index stations: ➊DEBE018 B Schöneberg-Belziger Straße ➋DEBE034 B Neukölln-Nansenstraße ➌DEBE064 B Neukölln-Karl-Marx-Straße 76 ➍DEBE065 B Friedrichshain-Frankfurter Allee ➎DEBE067 B Hardenbergplatz. Nine reference stations outside the low emission zone: ➏DEBE061 B Steglitz-Schildhornstraße ➐DEBE062 B Frohnau, Funkturm (not included in the figure since located approx. 13 km north of low emission zone) ➑DEBE063 B Neukölln-Silbersteinstraße) ➒DEBE066 B Karlshorst-Rheingoldstraße, no PM_10_ (not included in the figure since located approx. 5 km east of low emission zone) ➓ DEBE010 B Wedding-Amrumer Straße ⓫DEBE027 B Marienfelde-Schichauweg (not included in the figure since located approx. 8 km south of low emission zone) ⓬DEBE032 B Grunewald (not included in the figure since located approx. 4 km south west of low emission zone) ⓭DEBE051 B Buch (not included in the figure since located approx. 12 km north east of low emission zone) ⓮DEBE056 B Friedrichshagen (not included in the figure since located approx. 14 km south east of low emission zone).


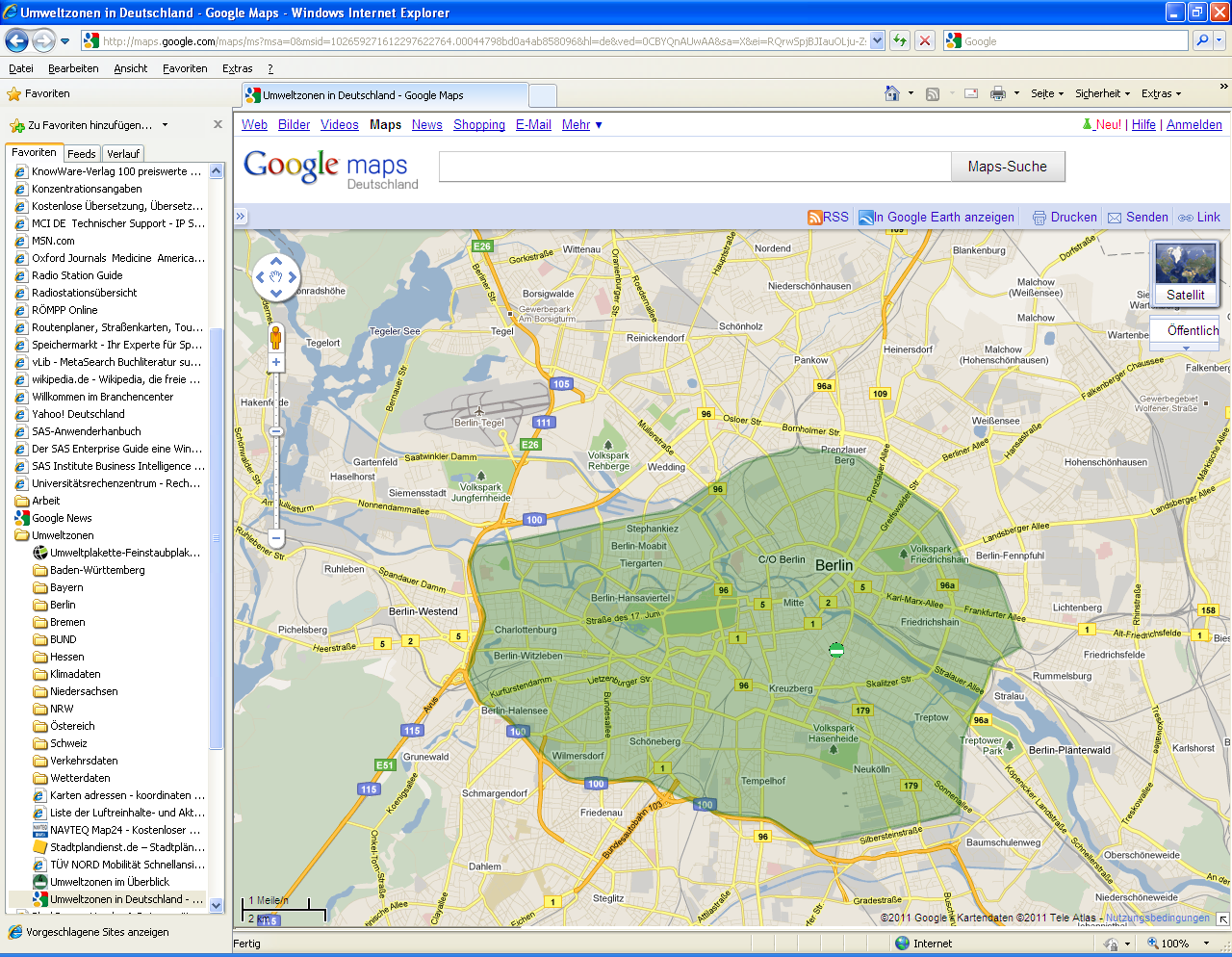


⓫

**➏**

**➋**

⓲

**➐**

**➎**

**➍**

**➌**

**➓**

⓴

**➊**

**➑**

**➒**

⓮

㉑

**Figure S11b:** Low emission zone **Berlin RUBIS-Messnetz** (marked area), implemented in 2008-01-01 (modified from www.map24.de). Ten index stations: ➊DEBE530 Hauptstraße 30 ➋DEBE504 Beusselstraße 66 ➌DEBE537 Alt Moabit 63 ➍DEBE545 Sonnenallee 68 ➎DEBE547 Landsberger Allee 6-8 ➏DEBE517 Neukölln-Nansenstraße ➐DEBE519 Friedrichshain-Frankfurter Allee ➑DEBE555 Herrmannplatz Laterne 21 ➒DEBE562 Friedrichstraße Laterne 156 ➓DEBE525 Leipziger Straße 32. Twelve reference stations outside the low emission zone:⓫DEBE501 Berliner Allee 118 ⓬DEBE577 Buch, no NO, no NO_x_ (not included in the figure since located approx. 12 km north of low emission zone) ⓭DEBE507 Grünauer Straße 4 (not included in the figure since located approx. 9 km south east of low emission zone) ⓮DEBE539 Schloßstraße 29 ⓯DEBE542 Tempelhofer Damm 148 ⓰DEBE513 Spreestraße 2 (not included in the figure since located approx. 5 km south east of low emission zone) ⓱DEBE514 Alt Friedrichsfelde 8a (not included in the figure since located approx. 3 km east of low emission zone) ⓲DEBE521 Steglitz-Schildhornstraße ⓳DEBE559 Buschkrugallee Laterne 3 ⓴DEBE522 Neukölln-Silbersteinstraße1 ㉑DEBE573 Badstraße ㉒DEBE576 Spandau, Klosterstraße 12 (not included in the figure since located approx. 6 km west of low emission zone).

**Hesse**


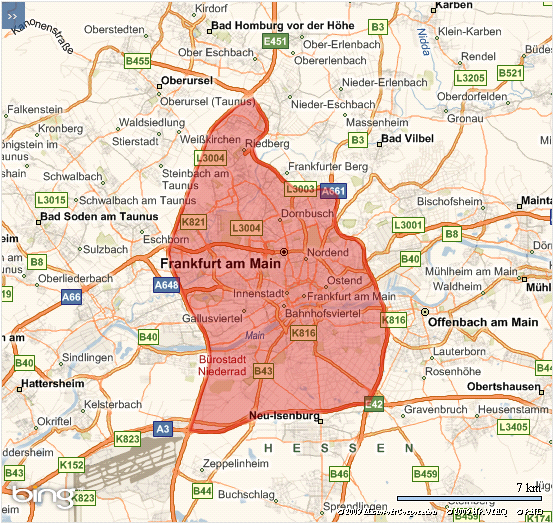


➊

➍

➋

**Figure S12:** Low emission zone **Frankfurt a.M.** (marked area), implemented in

2008-10-01 (modified from www.map24.de). One index station: ➊DEHE041 Frankfurt-Friedb.Ldstr. Three reference stations outside the low emission zone: ➋DEHE008 Frankfurt-Ost ➌DEHE011 Hanau (not included in the figure since located approx. 13 km east of low emission zone) ➍DEHE005 Frankfurt-Höchst.

**Lower Saxony**


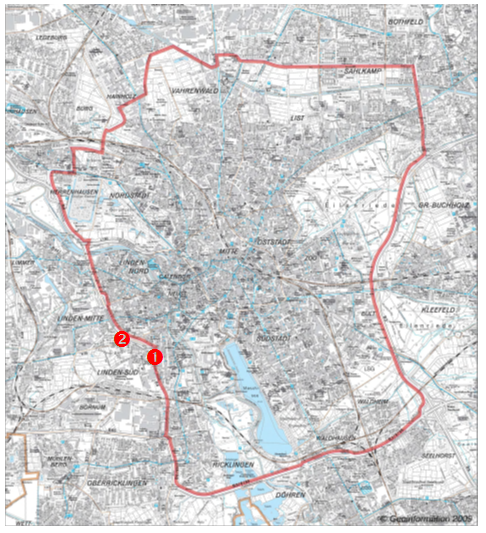


**Figure S13:** Low emission zone **Hannover** (marked area), implemented in

2008-01-01 (modified from www.map24.de). One index station: ➊DENI048 Hannover Verkehr. Four reference stations outside the low emission zone: ➋DENI054 Hannover ➌DENI011 Braunschweig, Broizemer Steinberg (not included in the figure since located approx. 49 km east of low emission zone) ➍DENI041 Weserbergland/ Rinteln, Brugfeldsweide (not included in the figure since located approx. 48 km south west of low emission zone) ➎DENI052 Allertal/Walsrode, Auf dem Kamp 8 (not included in the figure since located approx. 47 km north of low emission zone).

**North Rhine-Westphalia**


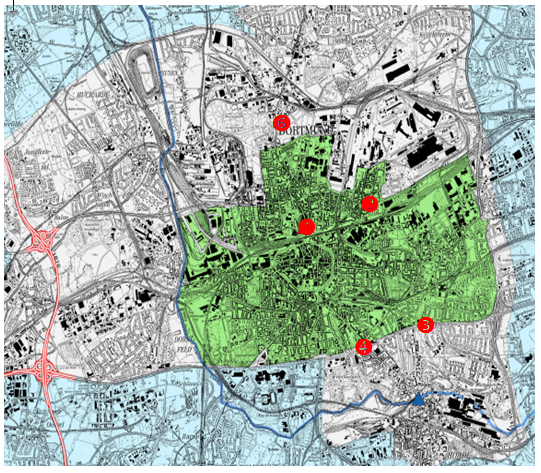


**Figure S14:** Low emission zone **Dortmund** (marked area), implemented in 2008-10-01, but Brackelerstr. 2008-01-01 (modified from www.map24.de). Four index stations: ➊DENW101 Steinstraße ➋DENW136 Brackeler Straße ➌DENW184 Westfalendamm 190, no NO, no NO_x_, no PM_10_ ➍DENW185 Rheinlanddamm 5-7, no NO, no NO_x_, no PM_10_. Four reference stations outside the low emission zone: ➎DENW002 Datteln-Hagem (not included in the figure since located approx. 15 km north west of low emission zone) ➏DENW008 Do-Eving ➐DENW029 Hattingen, An der Becke (not included in the figure since located approx. 19 km south west of low emission zone) ➑DENW179 Schwerte (not included in the figure since located approx. 8 km south of low emission zone).


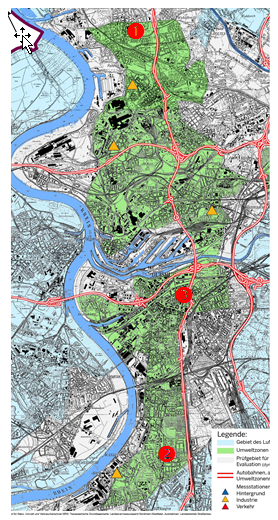


**Figure S15:** Low emission zone **Duisburg** (marked area), implemented in 2008-10-01 (modified from www.map24.de).Three index stations: ➊DENW034 Duisburg-Walsum ➋DENW040 Duisburg-Buchholz ➌DENW112 Kardinal-Galen-Straße. One reference station outside the low emission zone: ➍DENW038 45476 Mühlheim, Neustadtstraße (not included in the figure since located approx. 5 km east of low emission zone).


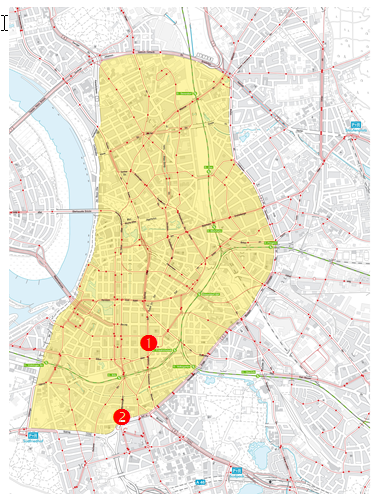


**Figure S16:** Low emission zone **Düsseldorf** (marked area), implemented in 2009-02-15 (modified from www.map24.de). Two index stations: ➊DENW082 Corneliusstraße ➋DENW216 Düsseldorf-Bilk, no NO, no NO_x_, no PM_10_. Four reference stations outside the low emission zone: ➌DENW042 Krefeld-Linn (not included in the figure since located approx. 14 km north west of low emission zone) ➍DENW071 Düsseldorf-Lörick (not included in the figure since located approx. 3 km west of low emission zone) ➎DENW078 Ratingen-Tiefenbroich (not included in the figure since located approx. 6 km north east of low emission zone) ➏DENW116 Krefeld Hafen (not included in the figure since located approx. 12 km north west of low emission zone).


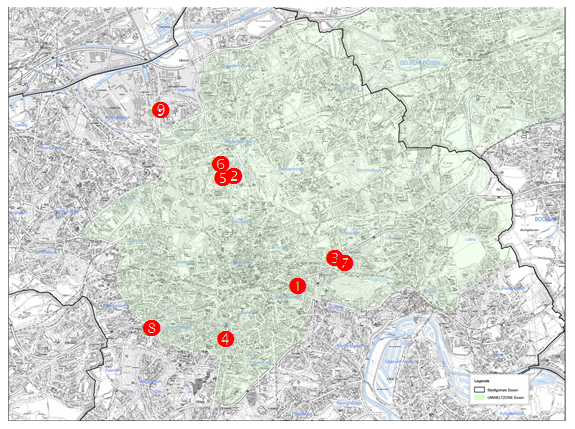


**Figure S17:** Low emission zone **Essen** (marked area), implemented in 2008-10-01 (modified from www.map24.de). Eight index stations: ➊DENW043 Ost Steeler Straße ➋DENW134 Gladbecker Straße ➌DENW135 Hombrucher Straße ➍DENW161 Alfredstraße 9/11, no NO, no NO_x_, no PM_10_ ➎DENW168 Gladbecker Straße 245, no NO, no NO_x_, no PM_10_ ➏DENW169 In der Baumschule 7, no NO, no NO_x_, no PM_10_ ➐DENW171 Hombrucherstraße 21/23, no NO, no NO_x_, no PM_10_ ➑DENW215 Hausackerstraße 11, no NO, no NO_x_, no PM_10_. Three reference stations outside the low emission zone: ➒DENW024 Essen-Vogelheim ➓DENW029 Hattingen-Blankenstein (not included in the figure since located approx. 10 km south east of low emission zone), ⓫ DENW162 Brückstraße 29, no NO, no NO_x_, no PM_10_ (not included in the figure since located approx. 4 km south of low emission zone).


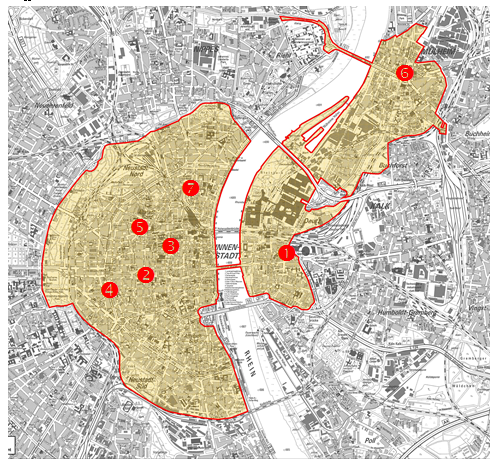


**Figure S18:** Low emission zone **Cologne** (marked area), implemented in 2008-01-01 (modified from www.map24.de). Seven index stations: ➊ DENW148 Justinianstraße 13-15, no NO, no NO_x_, no PM_10_ ➋DENW151 Neumarkt 25, no NO, no NO_x_, no PM_10_ ➌DENW153 Tunisstraße/ Elstergasse, no NO, no NO_x_, no PM_10_ ➍DENW164 Hohenstaufenring 57A, no NO, no NO_x_, no PM_10_ ➎DENW198 Gereonsdriesch 21, no NO, no NO_x_, no PM_10_ ➏DENW211 Clevischer Ring 3 ➐DENW212 Turiner Straße. Four reference stations outside the low emission zone: ➑DENW053 Cologne-Chorweiler (not included in the figure since located approx. 9 km north west of low emission zone) ➒DENW058 Hürth (not included in the figure since located approx. 7 km south west of low emission zone), ➓DENW059 Cologne-Rodenkirchen (not included in the figure since located approx. 4 km south of low emission zone), ⓫ DENW079 Leverkusen-Manfort (not included in the figure since located approx. 7 km north of low emission zone).


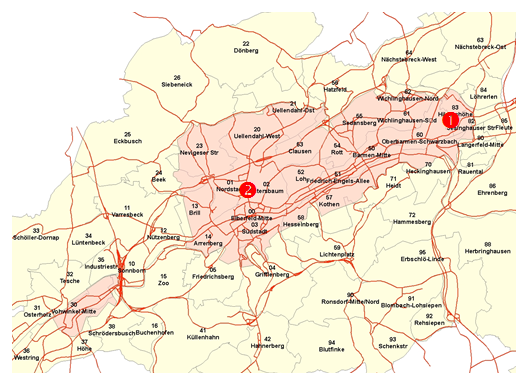


**Figure S19:** Low emission zone **Wuppertal** (marked area), implemented in 2009-02-15 (modified from www.map24.de). Two index stations: ➊DENW114 Wuppertal-Langerfeld, no NO, no NO_x_ ➋DENW189 Wuppertal Gathe. Two reference stations outside the low emission zone: ➌DENW029 Hattingen-Blankenstein (not included in the figure since located approx. 13 km north of low emission zone) ➍DENW080 Solingen-Wald (not included in the figure since located approx. 5 km south west of low emission zone).
